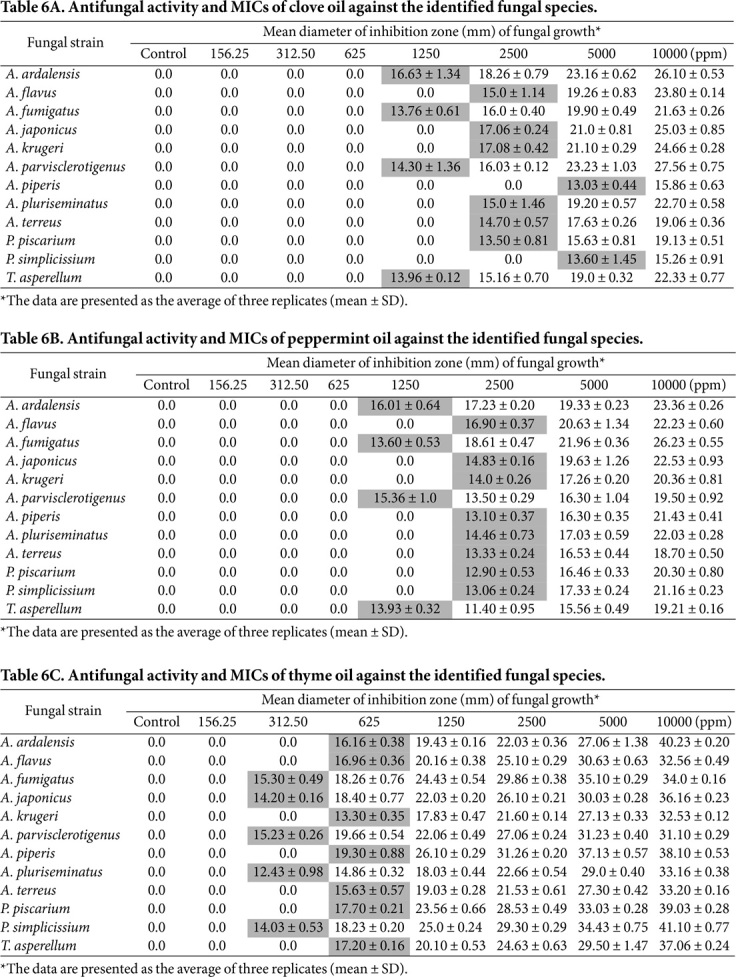# Natural Inhibitory Treatment of Fungi-Induced Deterioration of Carbonate and Cellulosic Ancient Monuments: Isolation, Identification and Simulation of Biogenic Deterioration

**DOI:** 10.4014/jmb.2404.04032

**Published:** 2024-08-30

**Authors:** Mohamed H. El-Sayed, Maha AlHarbi, Islam A. Elsehemy, Wafaa M. Haggag, Bahgat M. Refaat, Sharaf M. Ali, Amr Elkelish

**Affiliations:** 1 Department of Biology, College of Sciences and Arts-Rafha, Northern Border University, Arar, Saudi Arabia; 2 Department of Biology, College of Science, Princess Nourah bint Abdulrahman University, P.O. Box 84428, Riyadh11671, Saudi Arabia; 3 Department of Natural and Microbial Products Chemistry, Pharmaceutical Industry Division, National Research Centre, Dokki, Egypt; 4 Department of Plant Pathology, National Research Centre, Dokki, Egypt; 5 Department of Botany and Microbiology, Faculty of Science, Al-Azhar University, Cairo 11884, Egypt; 6 Central Research Laboratory, National Museum of Egyptian Civilization, Ministry of Antiquities, Cairo, Egypt; 7 Department of Biology, College of Science, Imam Mohammad Ibn Saud Islamic University, Riyadh, Kingdom of Saudi Arabia; 8 Botany Department, Faculty of Science, Suez Canal University, Ismailia 41522, Egypt

**Keywords:** Ancient monuments, NMEC, biogenic deterioration, *Aspergillus fumigatus*, thyme oil

## Abstract

Fungi play a significant role in the deterioration of various types of monuments. Therefore, the protection of ancient monuments from fungal attacks is an important goal that must attract the attention of researchers worldwide. A total of 69 fungal isolates were recovered from 22 deteriorated objects compromising paper, textiles, wood, and stone in the National Museum of Egyptian Civilization (NMEC) storeroom, Cairo, Egypt. The isolates were identified as 12 different species categorized into three different genera, namely, *Aspergillus* (9 species), *Penicillium* (2 species) and *Trichoderma* (1 species). Among them, *Aspergillus fumigatus* was the most prevalent species. Three essential oils were assessed for antifungal activity and compared with the antifungal effects of five synthetic microcides to identify a natural inhibitory treatment. Thyme oil and sodium azide were found to be the most active growth inhibitors, with minimum inhibitory concentrations (MICs) of 625 and 100 ppm, with inhibition zone diameters of 19.0 ± 0.70 – 23.76 ± 1.15 and 13.30 ± 0.35 – 19.66 ± 0.54 mm, respectively. An in vitro simulation of the biodeterioration process was conducted using spores of the *A. fumigatus* strain NMEC–PSTW.1 on model cubes made of paper, textile, wood, and stone materials. The changes in the characteristics of the artificially deteriorated materials were analyzed using environmental scanning electron microscopy/energy dispersive X-ray spectroscopy and Fourier transform infrared spectroscopy. The results revealed changes in the morphology, physical properties, and chemical composition induced by *A. fumigatus* NMEC–PSTW.1. Overall, thyme oil is recommended as a natural inhibitor to protect carbonate and cellulosic monuments in NMEC against fungal attack.

## Introduction

Cultural heritage plays a significant role in our lives, connecting individuals to their social values, religions, beliefs, and traditions. In addition, it reflects human identity and provides documentation of historical existence and activity [1].

The ancient Egyptian civilization, which extended along the Nile River, was considered one of the greatest civilizations for thousands of years (3300 BC to 525 BC) [2]. Ancient Egypt was considered the "state of stone" due to its reliance on stone as the primary raw material during the Pharaonic, Greco-Roman, and Arab periods [3]. The ancient Egyptians documented the details of their daily lives through carvings on temple walls, stones, clay, or papyri, creating a valuable repository of knowledge and learning for humanity as a whole [4]. Accordingly, the preservation of such ancient monuments is crucial to our understanding, as they are pivotal in comprehending human history and the evolution of civilizations.

The historical significance of ancient Egyptian monuments cannot be overstated since they are an enduring testimony to the rich cultural and artistic achievements of ancient civilizations. These monumental structures, such as the Pyramids of Giza, the Sphinx, and the Temples of Luxor, are considered valuable archaeological monuments in national museums. Furthermore, they represent extraordinary architectural and engineering achievements and offer valuable insights into ancient Egyptians' religious beliefs, customs, and daily life [5]. Therefore, the protection and restoration of these ancient Egyptian monuments are significant objectives that should attract scholars' interest worldwide, as they receive funding from many Egyptian and foreign organizations.

The National Museum of Egyptian Civilization, located in Cairo, the capital of Egypt, is considered the first of its kind in Egypt and the Arab world. It houses over 50,000 artifacts, making it one of the world's largest and most comprehensive collections of Egyptian artifacts. The museum showcases different phases of Egyptian civilization across various aspects of life and contains a large collection of Nubian monuments [6]. Despite the significance of the NMEC in preserving Egyptian cultural heritage sites, the spread of microbial infections has been observed in some archaeological objects, especially those housed in storage rooms [7, 8]. Archaeological objects found in the NMEC, particularly those composed of carbonate and cellulose, are highly susceptible to microbial biodeterioration because prevailing environmental conditions such as temperature and humidity are more suitable for microbial growth [9].

Biodeterioration was first defined by Hueck (1965 and 1968) as "any undesirable change in the properties of a material caused by the vital activities of organisms." This phenomenon can have irreversible effects on materials due to surface and internal changes [10]. Microorganisms, animals, and plants can biodegrade and deteriorate various types of archaeological materials through biochemical and biophysical mechanisms [11].

Biodeteriogenic microorganisms have the ability to utilize a substrate to support their growth and multiplication [11]. Consequently, environmental factors such as temperature, water availability, natural or artificial light exposure, and the material's properties, such as porosity, are crucial aspects to consider when understanding the mechanism of biodeterioration. The growth (biofilms) of microbial biodeteriogens can cause both mechanical and chemical transformations due to the microdecohesion and metabolic processes of the microorganisms and their secretions. Additionally, the accumulation of airborne pollutants in biofilms and corrode the substrate can contribute to these processes [12]. These effects can occur both on the surfaces and inside the material structure, leading to long-term deterioration and compromising the strength and durability of the monumental material [13].

Archeological artifacts are composed of various materials. Every material can be influenced to varying degrees, with carbonate and cellulosic monuments being particularly susceptible. In museums, archives, or library environments, numerous microorganisms can colonize paper, which can severely damage valuable documents. Therefore, protecting historical paper-based archaeology from microbial biodeterioration is essential for preserving cultural heritage [14]. Similarly, the stones used in archaeological or decorative objects provide an ideal environment for colonization by various microorganisms. The susceptibility of a stone to biological colonization caused by specific physical characteristics, including surface roughness and porosity, can result in aesthetic, physical, and chemical damage [15].

The deteriorating microorganisms that colonize archaeological materials include various species of fungi, certain algae, yeasts, cyanobacteria, and bacteria. The first colonization of organic nutrients by microorganisms, such as algae and cyanobacteria, is typically facilitated through photosynthesis. After invading the material, filamentous microorganisms generate biophysical attacks. Some microorganisms produce organic and inorganic acids, while other microbes release CO_2_, which is crucial for metabolic degradation [16]. In addition, microorganisms such as fungi can obtain various elements for their metabolism through the biosolubilization of some elements, such as aluminum, calcium, iron, potassium, and silicon. This process is known as microbial biosolubilization and involves the production of inorganic and organic acids through metabolic activities [17].

Fungi have a significant impact on the biodeterioration of different types of archaeological monuments due to their complex metabolic activities. A significant proportion of the global cultural heritage is threatened by fungal infestation. Fungi can deteriorate different substrates via different physical and chemical mechanisms. The process of hyphal growth and penetration into the substrate can lead to many symptoms, including discoloration, biopitting, cracking, exfoliation, and patina formation. Chemical mechanisms include the release of extracellular enzymes, pigment production, acid secretion, oxidation/reduction reactions, and the formation of secondary mycogenic minerals. These processes can lead to severe aesthetic and structural changes that are potentially irreversible, thereby permanently affecting artworks [18].

To control fungal biodeterioration in museums, temperature and humidity must be monitored to prevent fungal growth and keep the monuments clean and dust-free [19]. Furthermore, regular cleaning and microbiological monitoring are essential for preventing and treating fungal infestations. One issue in restoration is identifying deteriorating fungi and developing appropriate treatments for deteriorated monuments. Treatment can be mechanical [20], physical with UV and gamma rays [21], chemical [22], or biological with natural products and essential oils [23].

Using chemical fungicides has several disadvantages, *e.g.*, they are toxic to the environment, workers, and visitors. In addition, some chemical fungicides cause undesirable changes to archeological objects. Furthermore, the use of chemical fungicides requires special precautions, including museum closure for several days or even weeks [24]. Conversely, natural products and essential oils derived from plants might serve as excellent alternatives for this purpose. These products are safe for the environment, nontoxic, and do not require any special procedures [25]. Essential oils are concentrated hydrophobic liquids containing volatile secondary metabolites from aromatic plants [26]. Since the Middle Ages, they have been widely utilized for their fungicidal, bactericidal, antiparasitic, insecticidal, and other medicinal applications [24].

Many historical structures in Egypt are at risk of fungal deterioration. This persistent problem poses a significant threat to the preservation of these renowned sites. To preserve our cultural heritage for future generations, interdisciplinary research teams of microbiologists, chemists, archeologists, and ecologists should be involved in the challenging research opportunities related to heritage conservation. Therefore, the present study aimed to isolate and identify the fungal species responsible for the biodeterioration of some carbonate and cellulosic Egyptian monuments. In vitro experiments were conducted to assess the inhibitory effects of some natural and chemical fungicides. Furthermore, this study also examined the changes caused by the most deteriorating fungal species on the properties of artificially deteriorated objects.

## Materials and Methods

### Microbiological Media

The isolation and cultivation of deteriorating fungal species were carried out using various microbiological media, including Czapek–Dox agar (CZDA: 50.0 g/l, Sigma Aldrich, USA), malt extract agar (MEA: 41.83 g/l, HiMedia, India), potato dextrose agar (PDA: 39 g/l, Merck, Germany), and Sabourud dextrose agar (SDA: 65.0 g/l, LabMal, Malaysia). Other culture media used in this study were of high analytical grade.

### Chemicals and Solutions

All chemicals used in this study were of analytical grade and were used without further purification. The analytical standards of the synthetic microcides (cetrimonium bromide (CB): C_19_H_42_NBr, dichloroxylenol (DCX): C_8_H_8_Cl_2_O, para-chloro-meta-cresol (PCMC): C_7_H_7_ClO, sodium azide (SA): NaN_3_ and tetra-ethyl-ammonium-bromide (TEAB): (C_2_H_5_)_4_N(Br)), as well as the natural essential oils (clove, peppermint, and thyme), were all purchased from Sigma Aldrich. They were used to assess their growth inhibitory effects on deteriorating fungal species. The studied microcides were dissolved in ethyl alcohol (EA, 95%), while the essential oils were dissolved in dimethyl sulfoxide (DMSO, 5%). Both ethyl alcohol and dimethyl sulfoxide were purchased from Sigma Aldrich.

### Archaeological Objects

The archeological objects examined in this study (Fig. S1) were deteriorated monuments that showed visible microbial growth, patina and/or signs of microbial decay and were selected for sampling. A total of 22 deteriorated archaeological objects belonging to four different types, including papers (Holy Quran, *n* = 2), textiles (flax and wool around the mummy, flax fabric by fees, *n* = 5), woods (bed, coffin, small armoire and statues, *n* = 5) and stones (bowl and canopic jar, chert grinding stone, small flask, steles and statues, *n* = 10), were selected for sampling from the store of the NMEC (latitude 30.300.30°N, longitude 31.14.53°E), Egypt. The data of the analyzed objects, along with their types, numbers, and descriptions, are listed in Table S1.

### Sample Collection and Processing

A total of 58 samples, including 36 swabs, 10 needles, and 12 nondestructive surface scratches, were collected from the selected archaeological objects (Table S1). The samples collected from these objects were classified into four categories: paper (8 swabs), textiles (11 swabs), wood (5 swabs and 3 needles), and stone (12 swabs, 7 needles, and 12 nondestructive scratches). For the papers, textiles, and wooden monuments, three sterilized cotton swabs (amended with 0.1 ml of Tween 80) were swabbed from the surface (~1.0 cm^2^ each) of the infected area of each archaeological object. Each stone monument was nondestructively scraped three times from the infected area using a sterile scalpel. The collected scrapings were then kept in sterile Eppendorf tubes. In addition, three sterile needles (moistened with Tween 80) were used to pick fungal spores from the black spots or cavities present in both the wood and stone monuments.

To prevent the collected swabs and surface scrapings from drying, they were kept in an icebox at approximately 4°C and transported to the microbiology laboratory within one hour of collection [27]. In the laboratory, the samples were processed using three different techniques: the spread plate, the pour plate, and the dilution plate. Each sample was tested in triplicate.

### Isolation of Deteriorating Fungal Species

The sampled swabs were aseptically suspended in a tube containing 1.0 ml of saline solution (aqueous 0.45%NaCl). The tubes were shaken for 10 min using a programmable rotary mixer. By using the spread plate technique, 0.1 ml of each suspension was individually spread onto the surface of four different isolation media (CZDA, MEA, PDA, and SDA), with the addition of chloramphenicol (500 mg/l) to prevent the undesired bacterial growth [28].

The stone surface scratches were processed in two ways. First, using the pour plate technique, 0.1 g of each collected sample was placed individually in the center of a sterile plate and covered with autoclaved media (CZDA, MEA, PDA, and SDA). The plates were then thoroughly mixed with circular movements and left to solidify. Second, using the dilution plate technique, 0.1 g of each collected sample was suspended individually in 10.0 ml of sterile saline solution (aqueous 0.45% NaCl) in sterile Eppendorf tubes. After the suspensions were shaken for 15 min at room temperature on a rotary shaker at 150 rpm, they were serially diluted (10^−1^ to 10^−5^) with sterile saline. Subsequently, 0.1 ml of each dilution was inoculated individually onto the four media [29]. The collected needles were directly inoculated onto agar plates supplemented with the four media before being transported to the laboratory [16].

The plates that were inoculated using the aforementioned techniques were incubated at 25 ± 2°C for 4–7 days. They were then carefully examined daily. The grown fungal colonies were purified by subculturing on fresh agar plates of the same media using a hyphal tip and/or a single spore [30]. Afterward, the pure isolates were kept on the same isolation media (without antibiotics) slants at 4°C and stored in 20% glycerol under refrigerated conditions [31].

### Identification of the Fungal Isolates

**Morphological Characterization**

The pure fungal isolates obtained were subcultured on the same isolation media (CZDA, MEA, PDA, and SDA) and incubated at 25 ± 2°C for 3–7 days. The freshly grown cultures were characterized based on their macroscopic characteristics, such as colony diameter, growth pattern, appearance, reverse side, and pigmentation. The micromorphology of the vegetative and reproductive fungal structures was also characterized by microscopic examination using the tease-mount method of Pandya and Jain [29], with minor modifications. Briefly, mycelial fragments were pulled out with a sterile needle, and preparations were made with lactophenol cotton blue to observe septations, hyphal structures, and conidia or spores. The identified morphological characteristics were compared with the standard criteria [32]. Additionally, they were compared with the fungal database identification program of the Regional Center for Mycology and Biotechnology at Al-Azhar University, Cairo, Egypt. The comparison was done using an image analysis system mainly based on Soft Imaging GbH software (analy SIS Pro ver. 3.0).

### Molecular Identification

Molecular identification of the fungal isolates was based on amplification and sequencing of the internal transcribed spacer (ITS) region. The fungal genomic DNA was extracted according to the method of Diba *et al*.[33] with slight modifications. In brief, single colonies of each isolate were selected from three-day-old cultures plated on CZDA media. Subsequently, they were inoculated into a 250-ml Erlenmeyer flask containing 100 ml of CZD broth media, followed by incubation at 25 ± 2°C for 72 h. The freshly grown fungal mycelium mass was harvested from the liquid cultures, filtered, and then purified by washing with distilled H_2_O. The genomic DNA was extracted based on the phenol‒chloroform and glass bead method using a solution of lysis buffer containing SDS (1%), EDTA (1 mM), Tris-HCl (10 mM), NaCl (100 mM) and Triton X-100 (2%) in distilled water at pH 8.0. The extracted DNA was checked by 1.5% agarose gel electrophoresis [34].

The ITS gene region was amplified via polymerase chain reaction (PCR) using extracted DNA as a template and primers of ITS1 (5'-CTTGGTCATTTAGAGGAAGTAA-3') and ITS4 (5'-TCCTCCGCTTATT GATATGC-3')[35]. A total volume of 50 μl of the PCR mixture was prepared by mixing 5 μl of the DNA template with 45 μl of PCR buffer. The PCR buffer consisted of 20 mM Tris-HCl (pH 8.0), 50 mM KCl, 0.1 mM each of forward and reverse primers, and 1.5 U of Taq DNA polymerase. PCR was performed in a thermal cycler (XP Cycler, BIOER, China). The thermal program included initial DNA denaturation at 95°C for 5 min, followed by 30 cycles of denaturation at 95°C for 30 sec and annealing at 55°C for 30 sec. Subsequently, the extension was performed at 72°C for 1 min, with a final extension at 72°C for 5 min following the last cycle. DNA fragments were separated by electrophoresis in 1.5% agarose gels in Tris-borate-EDTA buffer (TBE) with 0.50 mg of ethidium bromide per ml. The obtained PCR products were sequenced using an ABI 3730x1 DNA sequencer at GATC Company (Germany) following previously published procedures.

Alignments of the obtained ITS sequences with those previously published in the National Center for Biotechnology Information (NCBI) database were performed using the GenBank search tool available on the Center's website BLAST (http://www.ncbi.nlm.nih.gov/BLAST). The phylogenetic tree was inferred using the neighbor-joining method with bootstrap testing (1,000 replicates). The evolutionary distances were computed using the Kimura 2-parameter method in MEGA11 software [36].

To further confirm the molecular identification of the deteriorating fungal species, *Aspergillus* and *Penicillium* species were subjected to sequencing of the β-tubulin (β-tub)-gene region using the primers of βtub F (5’-TGACGGGTGATTGGGATCTC-3’) and βtub R (5’-CGTCCGCTTCTTCCTTGTTT-3’) [37]. As for *Trichoderma* species they were subjected for sequencing of the translation elongation factor 1-alpha (TEF-1α) region using the primer EF1-728F (5'-CATCGAGAAGTTCGAGAAGG-3') and EF1-986R (5'TACTTGAAGGAACCCTTACC-3')[38].

The polymerase chain reaction (PCR) for amplification of the β-tub region was performed according to the method of Serrano *et al*. [37]. Briefly, the PCR mixture used consisted of 5 μl genomic DNA, 2.5 μl buffer, 0.5 μl dNTP, 3.6 μl MgCl_2_, 0.25 μl Fermantase-Taq polymerase and 0.5 μl of each primer. The mixture was incubated at 95°C for 5 minutes and amplification was performed for a total of 35 cycles as follows: Denaturation at 95°C for 1 min, annealing at 63°C for 90 sec, extension at 72°C for 2 min, and a final extension step of 10 min at 72°C.

As for the TEF-1α region, PCR amplification was performed according to the method of Druzhinina *et al*. [38]. Briefly 50 μL PCR reaction mixture consisting of PCR buffer (10×), Taq DNA polymerase (5U), triphosphate deoxynucleotide mix (dNTPs) (10 mM), MgCl_2_ (25 Mm), primers (10 μM) and DNA template 50 ng. The mixture was incubated at 95°C for 5 minutes and amplification was performed for a total of 35 cycles as follows: Denaturation at 95°C for 1 min, annealing at 58°C for 30 sec, extension at 72°C for 1 min, and a final extension step of 10 min at 72°C.

The products of multiplex PCR were electrophoresed in a 2% agarose gel with 0.1 g Saber Save dye, and the obtained sequences of β-tub and TEF-1α were compared with the public online database GenBank using the BLAST algorithm to determine sequence identity.

### Percentage of Species Frequency

The frequency of occurrence of the identified fungal species was calculated according to Gupta [39] using Eq. (1).

$$\text{Percent of frequency (PF) = }\frac{\text{Number of samples in which specific organisms occurred}}{\text{Total number of samples examined}}$$ (1)

The identified fungi were categorized into rare (0–25% frequency), occasional (26–50% frequency), frequent (51–75% frequency), or common (76–100% frequency) species based on the frequency of occurrence.

### Control of Monument-Deteriorating Fungi

**Preparation of Stock Solutions and Assayed Concentrations**

The antifungal activity and MICs of five synthetic microcides (CB, DCX, PCMC, SA, and TEAB) and three natural essential oils (clove, peppermint, and thyme) were evaluated against deteriorating fungal species. The microcides were prepared as stock solutions using the method described by Abdelhafez *et al*. [40]. In brief, 1.0 g of each microcide was separately dissolved in 1 L of 95% alcohol to obtain a concentration of 1,000 μg/ml. The stock solutions were diluted with alcohol using twofold serial dilutions to create gradient concentrations. Final concentrations of 25, 50, 100, 200, 400, and 800 ppm were obtained for each microcode.

For the essential oils, stock solutions were prepared according to the method of Puškárová *et al*. [41], with minor modifications. To determine the volume containing 1.0 g of each essential oil, the essential oils were individually weighed due to their varying densities. This amount was used in the tests at a full-strength (100%) concentration (stock solution, 1,000 μg/ml). Gradient concentrations were prepared using twofold serial dilution by diluting the stock solutions with 5% dimethyl sulfoxide. This resulted in final concentrations of 156, 312, 625, 1,250, 2,500, 5,000, and 10,000 ppm for each oil. The tests included controls consisting of a mixture of 95% pure alcohol and 5%DMSO to ensure that the solvent did not hinder microbial growth.

### In vitro Assays of Antifungal Activity and Determination of MICs

The fungal suspensions were prepared according to the modified method of El-Sayed *et al*. [42]. First, the tested fungal strains were subcultured on agar slants of isolation media (CZDA, MEA, PDA, and SDA) and then incubated at 25 ± 2°C for three days. Freshly prepared spore suspensions of these strains were obtained by washing the surface of the slant cultures with 1 ml of sterile saline (aqueous 0.45% NaCl solution) and shaking the suspensions for 5 min. Afterward, 100 μl of each spore suspension was used to inoculate a 500 ml Erlenmeyer flask with 250 ml of the corresponding isolation medium and mixed well. After the inoculated media were poured into sterile Petri plates (90 mm diameter), wells (6 mm) were drilled into the seeded plates using a sterile cork borer. Then, 100 μl of each microcide/essential oil concentration was added individually to each well. Each microcide and essential oil was tested at 100% strength (original concentration without dilution) and various concentrations ranging from 25–800 ppm in 95% alcohol and 156–1,000 ppm in 5% DMSO. One well with pure 95% alcohol without microcide and one with pure 5% DMSO without essential oil were used as controls. The inoculated plates were left at 4°C for 2 h and incubated at 25 ± 2°C for five days. The antifungal tests were performed in triplicate, and the resulting zone of inhibition (ZI) diameter was measured (mm) and recorded. In addition, the MIC of each microcide/essential oil was determined.

### Preparation of Model Cubes and Simulation of the Artificial Deterioration Process

Model cubes (5 cm^3^) of commercial paper, stone, textile, and wood materials obtained from local markets were used as short-term simulation models for the artificial in vitro deterioration process according to the method of ElBaghdady *et al*. [8], with minor modifications. In brief, the predominant fungus, *Aspergillus fumigatus*, was subcultured on PDA plates at 25 ± 2°C for four days. After that, three loopfuls of the cultured spores were suspended individually in 10.0 ml of sterile saline (aqueous 0.45% NaCl) solution. The turbidity was adjusted to obtain a suspension of approximately 10^5^–10^6^ spores/ml. One milliliter of the spore suspension was evenly distributed on each cube in a sterile beaker under septic conditions. The beaker was then incubated at 25 ± 2°C for 90 days. The control (noninoculated) cubes were treated with 0.45% NaCl. The inoculated cubes were visually examined weekly for 7, 15, 30, 45, 60, and 90 days to detect signs of biogenic fungal deterioration. The physical, morphological, and chemical properties of the cubes were examined to identify indicators of deterioration. This was done using physical and chemical analyses.

### Analysis of the Simulated Material Properties

**Analysis of the physical and mechanical properties.** The physical and mechanical properties, including water absorption, density, porosity, and mechanical strength, were measured for the artificially deteriorated cubes using Eqs. (2–5) [43].

$$\text{Water absorption (\%)}=\frac{\text{Wet weight-Dry weight}}{\text{Dry weight}}\times 100$$ (2)

$$\text{Density=}\frac{\text{Weight}}{\text{Length×Width×Height}}{\text{=Kg/cm}}^{\text{3}}$$ (3)

Porosity (%) = Density×Water absorption (4)

$$\text{Compressive strength = }\frac{\text{Mechanical load}\times 102}{\text{Length}\times \text{Width}}={\text{Kg/cm}}^{3}$$ (5)

All aforementioned parameters were calculated as percentages of reduction compared with the control.

### Analysis of the Morphological Properties

The changes in the macromorphology of the surfaces of all the artificially deteriorated cubes were examined visually. The changes in the surface of the artificially deteriorated stone cubes were examined using an environmental scanning electron microscope (ESEM) in combination with electron dispersive X-ray spectroscopy (EDXS) (ESEM- EDXS, JEOL-JSM5500 L V, Japan).

### Analysis of Chemical Properties and Mineral Constituents

The chemical composition of the artificially deteriorated cubes was analyzed using energy-dispersive X-ray spectroscopy (EDXS) on a scanning electron microscope and Fourier transform infrared (FTIR) spectroscopy [44].

### Data Analysis

The data collected in all experiments are the mean values of three independent replicates. The data were subjected to analysis of variance (ANOVA) using the statistical package SPSS v17. The comparisons between treatments were analyzed using the Tukey HSD test at a significance level of *p* ≤ 0.05.

## Results

### Isolation of the Deteriorating Fungi

Using our screening process to identify fungal species that cause deterioration in archaeological objects at the NMEC, we obtained a total of 69 pure fungal isolates (NMEC1–NMEC69) using various cultivation media. These isolates were recovered from a total of 58 different samples collected from 22 deteriorated archaeological objects. The samples were classified into four categories: paper (8 samples), wood (8 samples), textiles (11 samples), and stone (31 samples). Since the samples were only taken from the infected (deteriorated) areas of selected archaeological objects, all the collected samples were positive for colonizing fungi. Therefore, the total number of fungal isolates found (69) was distributed across all the collected samples.

The correlation between the number of fungal isolates obtained and the number of samples collected (Fig. S2) was relatively high (*r* = 0.83), as the number of isolates in stone, textile, and wooden objects increased with the number of samples taken. However, the number of isolates associated with the paper objects was significantly higher than that of the corresponding wooden objects, even though an equal number of samples were collected.

**Fig. 1:**
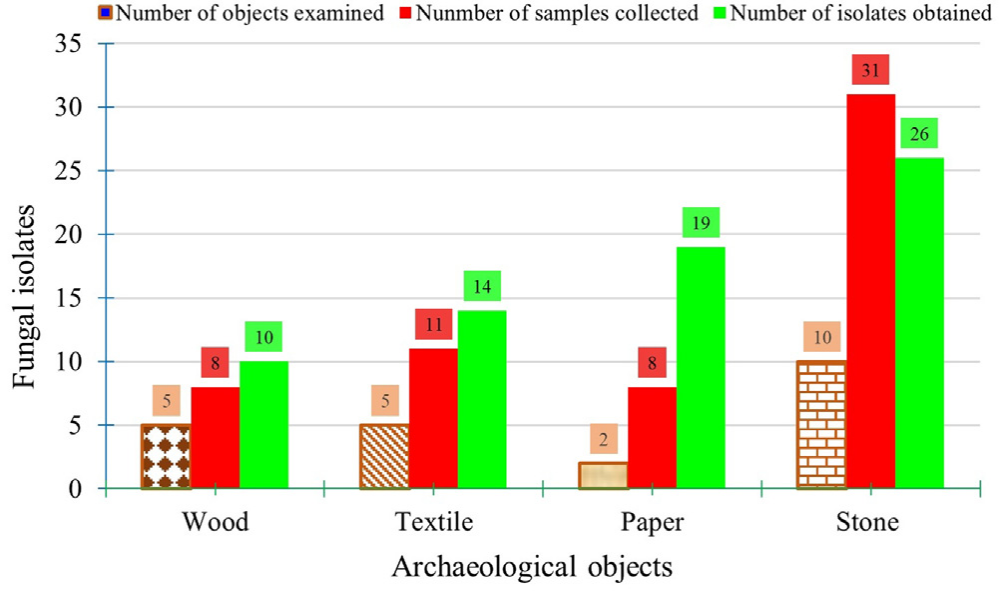
Distribution of fungal isolates among the examined archaeological objects.

The distribution of the recovered isolates varied based on the type of archaeological objects isolated from, the number of samples collected, the sampling method, and the recovery media used. In brief, most fungal isolates were recovered from stones (*n* = 26, 37.68%), followed by paper (*n* = 19, 27.53%) and textiles (*n* = 14, 20.28%). Conversely, the wooden monuments had the lowest number of fungal isolates (Fig. 1). Their distribution according to the sampling method was as follows: swabbing (*n* = 56, 81.15%), followed by needling (*n* = 8, 11.59%) and scraping (*n* = 5, 7.24%). Regarding the distribution by isolation media, they were categorized as CZDA (*n* = 43, 62.31%) and PDA (*n* = 24, 34.78%). The remaining isolates (*n* = 2, 2.89%) were obtained on all media used. The recovered fungal isolates' data and their distribution among the selected archaeological objects, the isolation media, and their growth characteristics are depicted in Table S2.

### Identification of Isolated Fungal Species

**Fig. 2:**
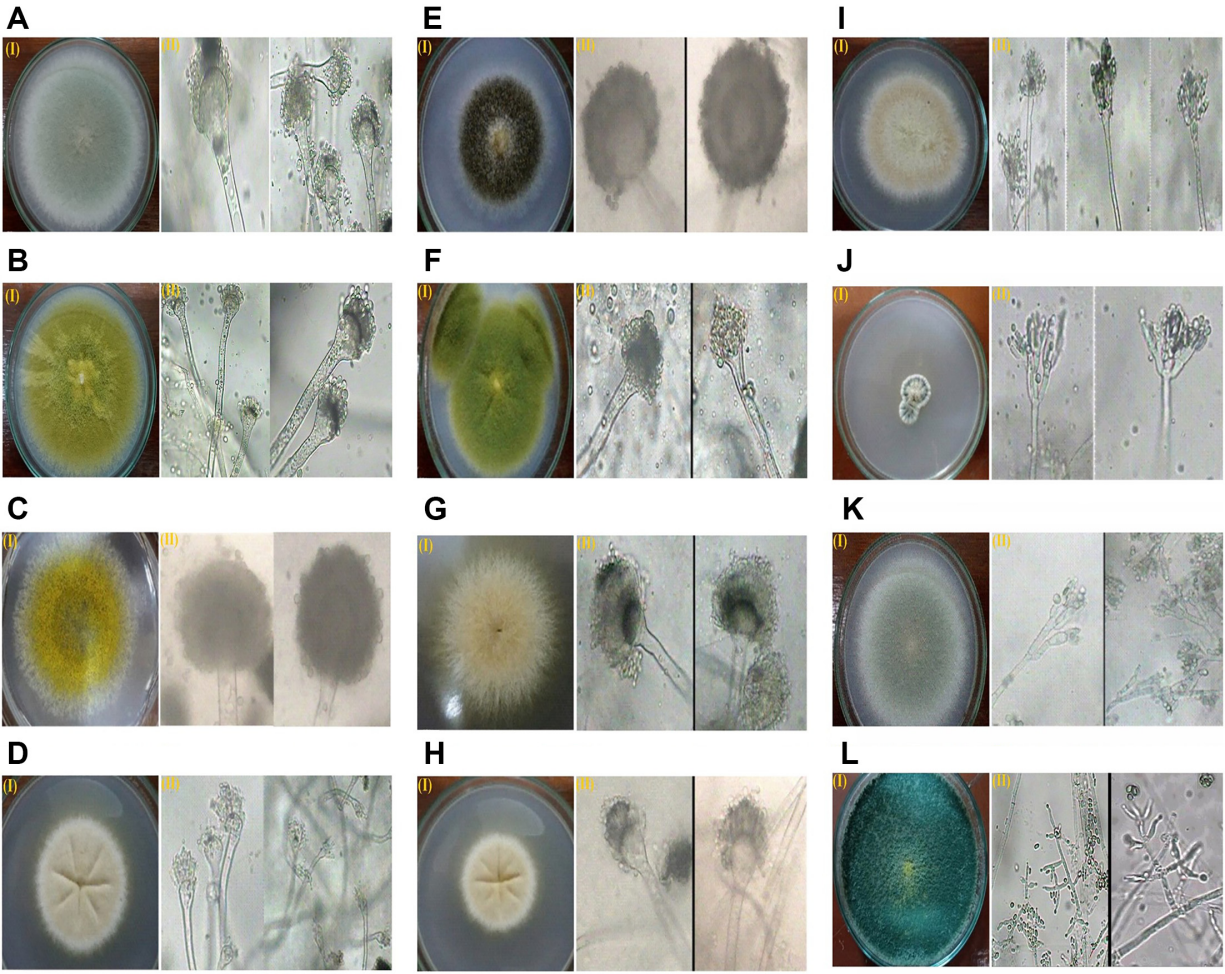
The identified fungal species recovered from the deteriorated archaeological objects under study. (**A**) *A. fumigatus*, (**B**) *A. flavus*, (**C**) *A. oryzae*, (**D**) *A. flavipes*, (**E**) *A. japonisas*, (**F**) *A. parasiticus*, (**G**) *A. terreus*, (**H**) *A. aureus*, (**I**) *A. unguis*, (**J**) *P. simplicissium*, (**K**) *P. canescens*, and (**L**) *T. harzianum*. In this figure, the right photograph (I) shows the macroscopic characteristics of the culture features, while the left photograph (II) shows the micromorphology of the vegetative and reproductive fungal structures.

**Morphological identification.** The recovered sixty-nine isolates were identified based on macroscopic examination of their colony and culture characteristics and microscopic examination of their vegetative and reproductive structures. The results are shown in Table S3A–S3L and Fig. 2A–2L). There were 12 different species belonging to three different genera: *Aspergillus* (*n* = 9 species, 75.0%), *Penicillium* (*n* = 2 species, 16.66%), and *Trichoderma* (*n* = 1 species, 8.33%).

The identified species of the genus *Aspergillus* included *A. fumigatus* (*n* = 14 isolates, 20.28%), *A. flavus* (*n* = 11 isolates, 15.94%), *A. oryzae* (*n* = 8 isolates, 11.59%), *A. flavipes* (*n* = 6 isolates, 8.69%), *A. japonisas* (*n* = 5 isolates, 7.24%), *A. parasiticus* (*n* = 4 isolates, 5.79%), *A. terreus* (*n* = 4 isolates, 5.79%), *A. aureus* (*n* = 4 isolates, 5.79%), and *A. unguis* (*n* = 3 isolates, 4.34%) (Fig. 2A–2I). The identified *Penicillium* ssp. included *P. simplicissium* (*n* = 6 isolates, 8.69%) and *P. canescens* (*n* = 2 isolates, 2.89%) (Fig. 2J, 2K), whereas the genus *Trichoderma* included only one species of *T. harzianum* (*n* = 1, 1.44%) (Fig. 2L).

Morphological identification revealed that the fungal isolates NMEC–P1, P4, P9, P18, T25, T27, W40, W42, S49, S51, S54, S61, S64 and S67 were *A. fumigatus*; the isolates NMEC–P3, P6, P10, T22, T23, W38, S57, S58, S60, S62, and S68 were *A. flavus*; the isolates NMEC–P5, P12, T24, T32, W39, S44, S46 and S55 were *A. oryzae*; the isolates NMEC–P7, P13, T33, W37, W41, and S47 were *A. flavipes*; the isolates NMEC–P8, T20, T26, T31, and W36 were *A. japonisas*; the isolates NMEC–P11, P17, T28, and W43 were *A. parasiticus*; the isolates NMEC–T30, S48, S52 and S66 were *A. terreus*; the isolates NMEC–W35, S50, S56 and S65 were *A. aureus*; the isolates NMEC–P15, S59, and S69 were *A. unguis*; the isolates NMEC–P16, T21, W34, S45, S53, and S63 were *P. simplicissium*; the isolates NMEC–P4 and P19 were *P. canescens*, whereas the isolates NMEC–P2 and T29 were *Trichoderma harzianum*.

### Molecular Identification

**Fig. 3:**
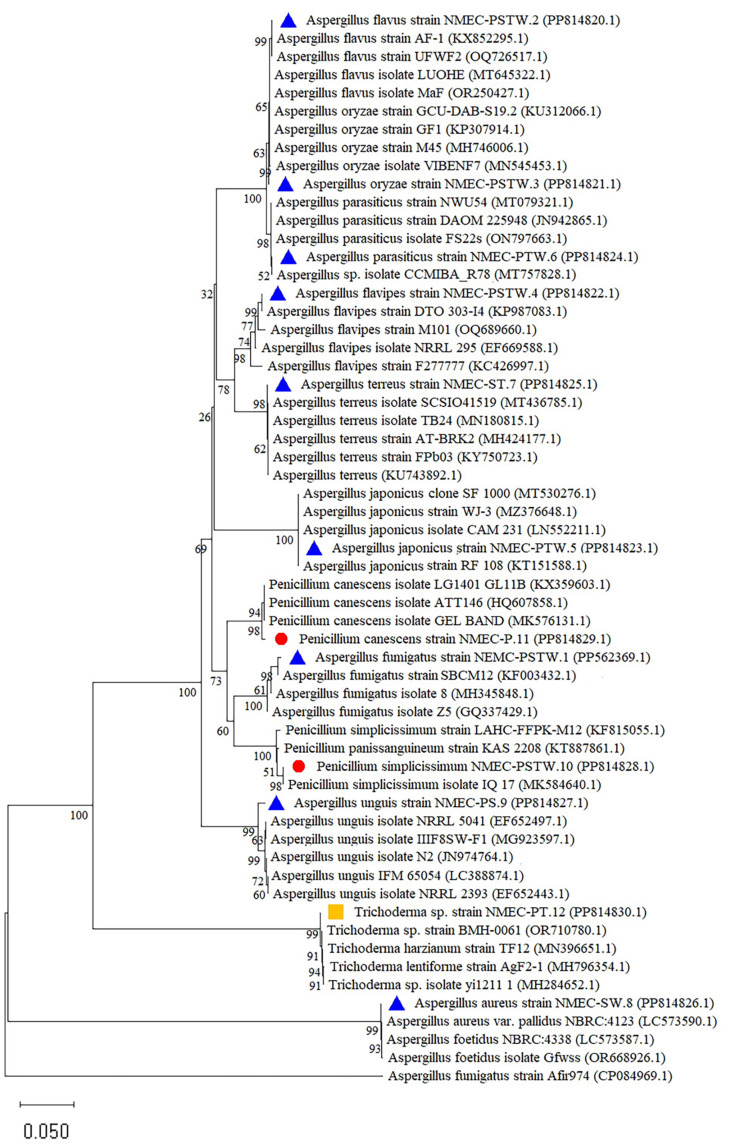
The phylogenetic tree of the ITS sequences of the twelve deteriorating fungal species and corresponding reference sequences retrieved from NCBI was inferred using the neighbor-joining method and Kimura 2-parameter phylogenetic analysis in MEGA 11.0 software.

The morphological identification of the obtained fungal species was confirmed by the molecular phylogeny of the partial sequence of the ITS gene (accession numbers PP562369 and PP814820–PP814830). The ITS sequence analysis identified these 12 fungal species as *Aspergillus fumigatus* strain NMEC–PSTW.1, *Aspergillus flavus* strain NMEC–PSTW.2, *Aspergillus oryzae* strain NMEC–PSTW.3, *Aspergillus flavipes* strain NMEC–PSTW.4, *Aspergillus japonicus* strain NMEC–PTW.5, *Aspergillus krugeri* strain NMEC–PTW.6, *Aspergillus terreus* strain NMEC–ST.7, *Aspergillus aureus* strain NMEC–SW.8, *Aspergillus unguis* strain NMEC-PS.9, *Penicillium simplicissimum* strain NMEC–PSTW.10, *Penicillium canescens* strain NMEC–P.11, and *Trichoderma* sp. strain NMEC–PT.12. BLAST analysis of these fungal strains revealed 98.38–99.81% identity (Table 1) with the ITS sequences of related species (Fig. 3).

### BLAST Analysis of the β-tub and TEF-1α Gene Regions

**Fig. 4:**
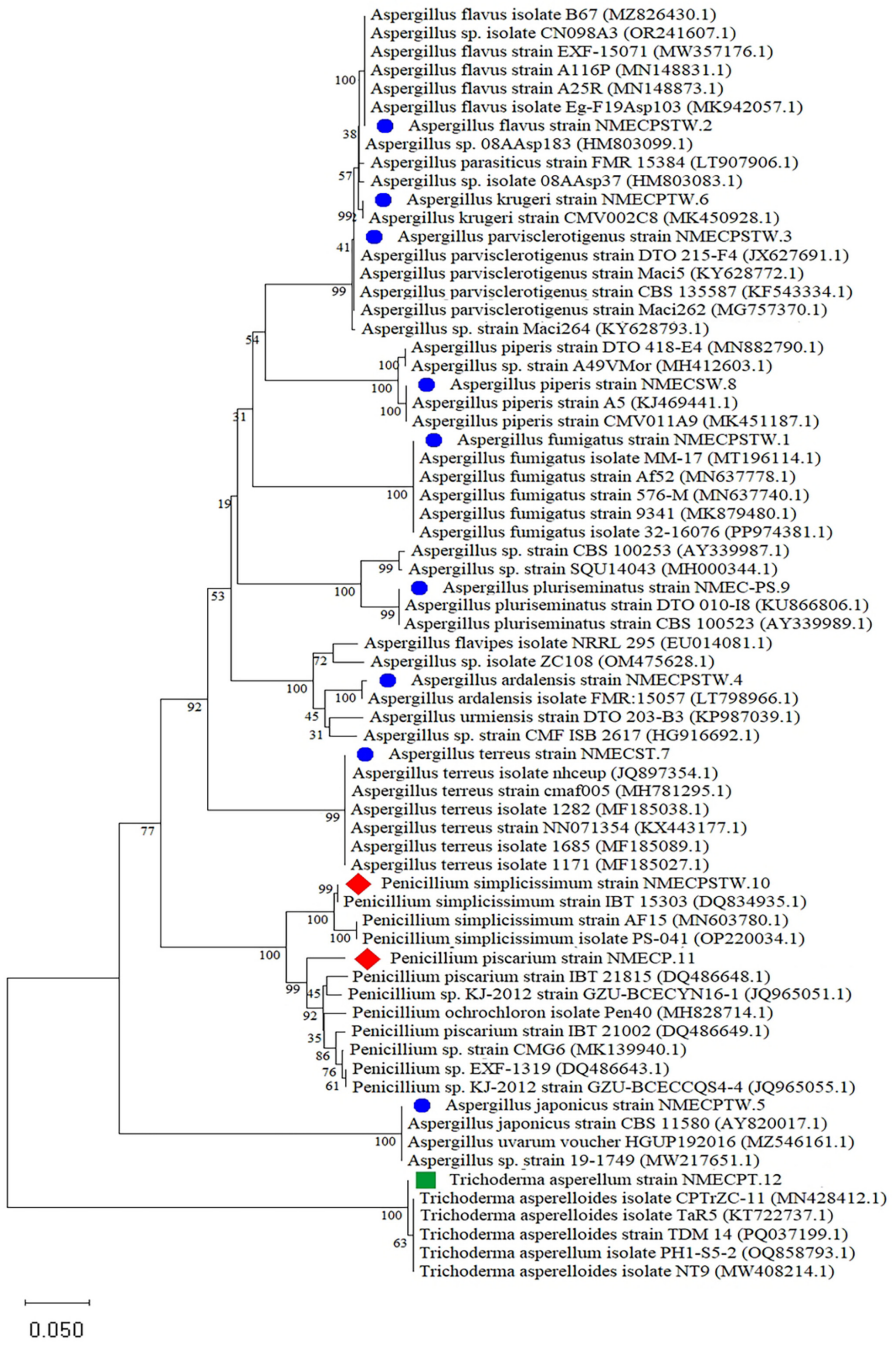
Phylogenetic tree of the twelve deteriorating fungal species using NCBI analysis of the β-tub and TEF- 1α regions inferred using the neighbour-joining method and Kimura's 2-parameter phylogenetic analysis in MEGA 11.0 software.

The NCBI BLAST database was used to identify the deteriorating fungal strains based on the multi-locus gene sequences of β-tub and TEF-1α. The results of BLAST analysis of these fungal strains showed 99.33–100.0%identity (Table 2) with the corresponding gene sequences of related species (Fig. 4).

Table 2 contains the species names and the accession numbers of the database hits. All strains displayed varying percentages of the maximum identity and E- values. Most strains provided “best hits” with similarity values between 99.0–100.0 %.

Based on the isolation source, the fungal species identified were found to be randomly distributed among the deteriorated archaeological objects. The analysis of *Aspergillus* spp. distribution (Table 3A) shows that *A. fumigatus*, *A. flavus*, *A. parvisclerotigenus*, and *A. ardalensis* were distributed on all archaeological objects examined, as their percentage frequency was 100%, so they were categorized as a common frequency class. *A. japonisas* and *A. krugeri* did not colonize the stone. The frequency percentage was 75%, so they were classified as a frequent frequency class. In contrast, *A. terreus* did not colonize paper or wood, and *A. piperis* did not colonize paper or textiles. In addition, *A. pluriseminatus*, which did not colonize textiles or wood, was classified as an occasional pathogen, with a frequency percentage of 50%.

The distributions of the identified *Penicillium* and *Trichoderma* ssp. Table 3B indicates that *P. simplicissium* colonized all archeologies, as its percentage frequency was 100%. Therefore, it was categorized as a common frequency class. *Trichoderma asperellum*, which colonized only paper and textile objects, was classified as an occasional pathogen with a frequency percentage of 50%. *P. piscarium*, which colonized only paper objects, was classified as rare, with a frequency percentage of 25%. The occurrence, percentage frequency, and frequency class of the identified species are summarized in Table 4.

According to the data obtained, *A. fumigatus* was shown to be one of the most prevalent species, with a total of 14 isolates. It was found to colonize all of the archeological objects examined, with varying percentages. The stone artifacts had the highest number of isolates, accounting for 86.96% of the total. Paper objects had the second highest number of isolates, accounting for 57.97%. Nevertheless, wooden and textile artifacts each contained 28.99% (Fig. S3). Therefore, it was designated *A. fumigatus* strain NMEC–PSTW.1 to identify the objects colonized by it and was selected for further studies.

### Inhibitory Treatment of Monument-Deteriorating Fungi

**In vitro antifungal activity of microcides and determination of MIC.** In this study, the effects of five synthetic microcides, CB, DCX, PCMC, SA, and TEAB, at concentrations ranging from 25 to 800 ppm (Table 5A–5D) and three natural essential oils, clove, peppermint, and thyme oil, at concentrations ranging from 156 to 10000 ppm (Table 6A–6C), on the isolated fungal strains were tested in vitro. All the synthetic microcides showed variable inhibitory growth activities with different MICs, except for PCMC, which was inactive at any tested concentrations. The CB results (Table 5A) indicated no inhibition at concentrations of 25 and 50 ppm. However, all species were inhibited at a concentration of 100 ppm, with a mean ZI diameter of 14.0 ± 0.40 to 23.10 ± 0.53 mm, except for *A. pluriseminatus*, which was inhibited at 200 ppm, with a ZI diameter of 13.30 ± 0.43 mm. Therefore, 100 ppm CB was the MIC that inhibited all fungal species tested, except for *A. pluriseminatus*, whose MIC was detected at 200 ppm.

DCX was only active against all treated fungal species at a concentration of 800 ppm and exhibited ZI diameters ranging from 12.60 ± 0.45 to 17.10 ± 1.30 mm. No inhibition was observed at the lower concentrations (25-400)(Table 5B). Consequently, the MIC of this microcide was 800 ppm. SA showed no inhibition at concentrations of 25 ppm and 50 ppm. However, all the isolates were inhibited at a concentration of 100 ppm, with ZI diameters ranging from 19.0 ± 0.70 to 23.76 ± 1.15 mm (Table 5C). Consequently, the MIC of SA is 100 ppm. Concerning TEAB, no inhibition was observed at concentrations between 25 ppm and 200 ppm. Conversely, all isolates were inhibited at a concentration of 400 ppm, with ZI diameters ranging from 14.06 ± 0.32 to 19.30 ± 0.88 mm (Table 5D). Therefore, 400 ppm TEAB is the MIC that inhibits all fungal species tested.

Clove oil (Table 6A) showed no inhibition at concentrations ranging from 156 ppm to 625 ppm. Some species, including *A. ardalensis*, *A. fumigatus*, *A. parvisclerotigenus*, and *Trichoderma asperellum*, were inhibited at a concentration of 1250 ppm, with the mean diameter of the ZI ranging from 13.76 ± 0.61 to 16.63 ± 1.34 mm, while other species, such as *A. flavus*, *A. japonicus*, *A. krugeri*, *A. terreus*, *A. pluriseminatus*, and *P. canescens*, were inhibited at a concentration of 2500 ppm, with a ZI diameter between 13.50 ± 0.81 and 18.26 ± 0.79 mm, while *A. piperis* and *P. simplicissium* were inhibited at a concentration of 5000 ppm, with a ZI diameter of 13.03 ± 0.44 and 13.60 ± 1.45 mm, respectively. Thus, the MIC of clove oil was detected at concentrations of 1250 and 2500 ppm.

For peppermint oil (Table 6B), no inhibition was detected at concentrations of 156.25, 312.50, and 625 ppm, while three species, *A. ardalensis*, *A. fumigatus*, *A. parvisclerotigenus*, and *Trichoderma* sp., were inhibited at a concentration of 1250 ppm, with a ZI diameter of 16.01 ± 0.64 - 13.60 ± 0.53 mm. The other species were inhibited at a concentration of 2500 ppm, with the mean diameter of the zone of inhibition ranging from 12.90 ± 0.53 to 16.90 ± 0.37 mm.

The results of thyme oil (Table 6C) showed that no inhibition was observed at a concentration of 156.25 ppm; five species, including *A. fumigatus*, *A. japonicus*, *A. parvisclerotigenus*, *A. pluriseminatus*, and *P. simplicissium*, were inhibited at a concentration of 312.50 ppm, with a ZI diameter of 12.43 ± 0.98 - 15.30 ± 0.49 mm. The remaining species were inhibited at a concentration of 625 ppm, with ZI diameters ranging from 13.30 ± 0.35 to 19.30 ± 0.88 mm.

Since each of the tested microcides had a different effect on each fungal strain, it was necessary to determine the ideal MIC for each fungal strain. These data can be used to recommend the best concentration for the biotreatment of infected monuments, and the data on the ideal MICs of different microcides are shown in Table S4A-S4B.

### Simulation of the Deterioration Process

The aged samples of paper, textile, wood, and stone materials artificially deteriorated in vitro with a spore suspension of *A. fumigatus* NMEC-PSTW.1, which was allowed to grow on these materials at 28°C for 90 days. The samples were examined regularly every 15 days over 90 days for signs of biological deterioration and then compared with the (noninoculated) control samples. At the end of the incubation period, the properties of the samples were analyzed physically, morphologically, and chemically.

**Fig. 5:**
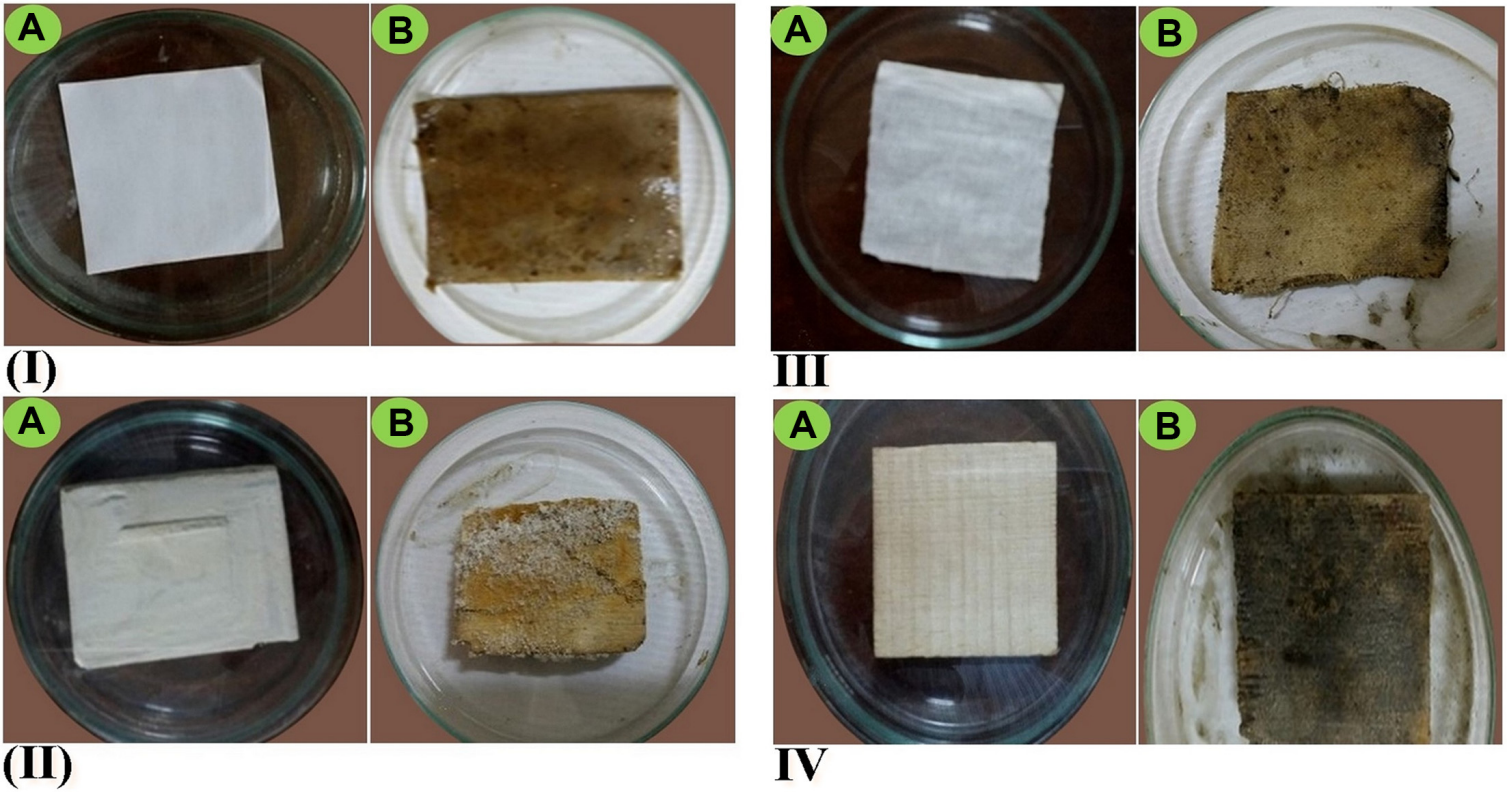
Biodeterioration signs caused by spores of *A. fumigatus* NMEC-PSTW.1 for model cubes of different materials: (I) paper, (II) stone, (III) textile, and (IV) wood. In this figure, the left photograph (**A**) is the control (noncolonized), and the right (**B**) is the treated (colonized) cube.

Due to fungal growth, the infected cubes showed different signs of visual deterioration (Fig. 5), including color changes and black spots on the paper cubes (Fig. 5I), pigmentation and gray decay spots on the stone cubes (Fig. 5II), color changes, the appearance of black spots and changes in feel from smooth to rough on the textile cubes (Fig. 5III), and gray‒black decay spots and black pigmented spots on the wooden cubes (Fig. 5IV).

### Analysis of the Physical Changes in the Simulated Cubes

Examination of the artificially deteriorated cubes revealed various physical changes in the different materials infected with *A. fumigatus* NMEC-PSTW.1. The porosities of the paper, stone, textile, and wood were reduced by 40.40 ± 2.12%, 38.43 ± 1.35%, 33.86 ± 2.86%, and 48.56 ± 3.10%, respectively. The strength was also reduced by 55.99 ± 2.40%, 29.96 ± 1.45%, 26.15 ± 2.23%, and 46.16 ± 2.24% for paper, stone, textile, and wood, respectively (Fig. S4).

### Analysis of the Morphological Changes in the Simulated Cubes

**Fig. 6:**
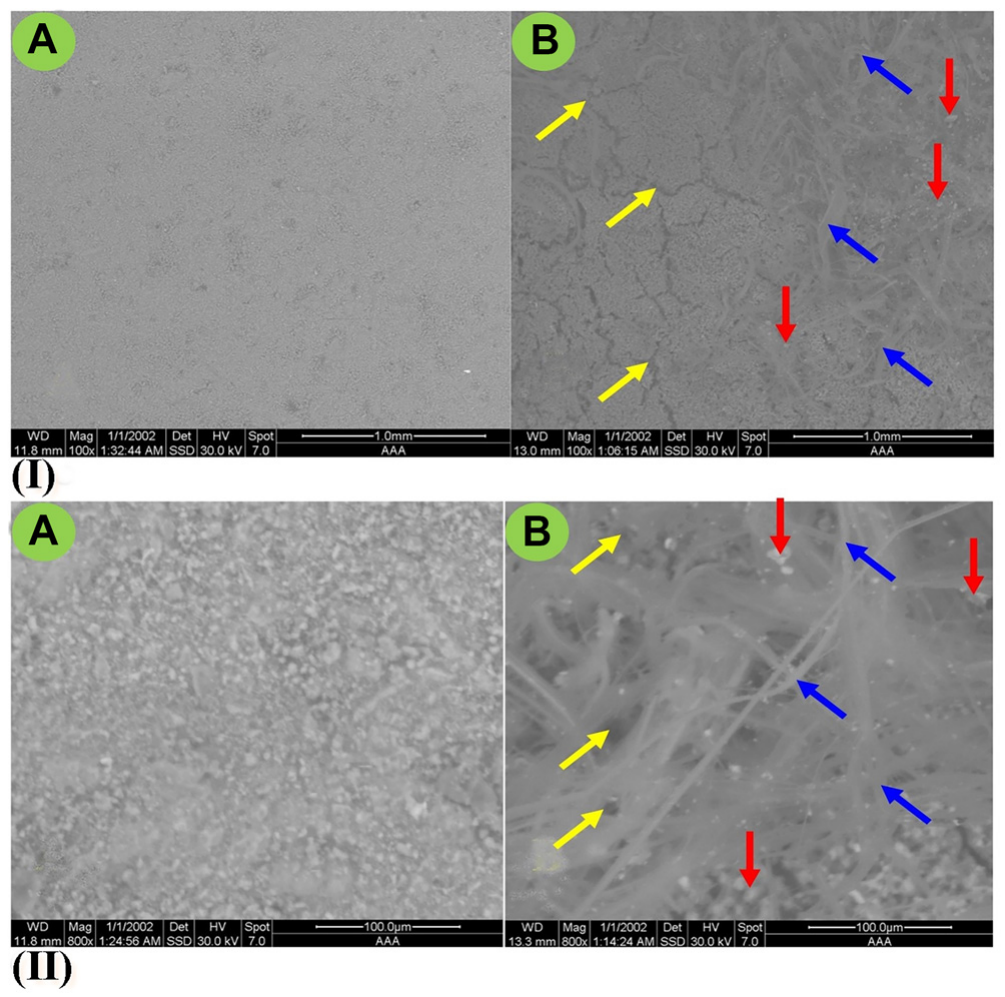
ESEM micrographs of stone cubes. (**A**) noncolonized stone (control), (**B**) stone inoculated with *A. fumigatus* NMEC-PSTW.1; (I) magnification scale 100× (scale bar =1 mm); (II) magnification scale 800× (scale bar = 100 μm). In this figure, the signs of deterioration are marked by yellow arrows (for surface cracking), blue arrows (for the fungal hyphae within limestone granules), and red arrows (for the fungal biofilm and exopolymeric substances).

The changes in the micromorphological properties of the artificially deteriorated stone cubes were visualized in the different ESEM images (Fig. 6), which showed a wide range of deterioration signs. The physical distortion appeared in the form of cracking (voids or pits) between the grains, while the uncolonized limestone (control) showed normal amorphous grains. In addition, the mycelia of *A. fumigatus* NMEC-PSTW.1 were distributed around the grains, and a microbial biofilm and exopolymeric substances were also found.

### Analysis of the Chemical Changes in the Simulated Cubes

**Fig. 7:**
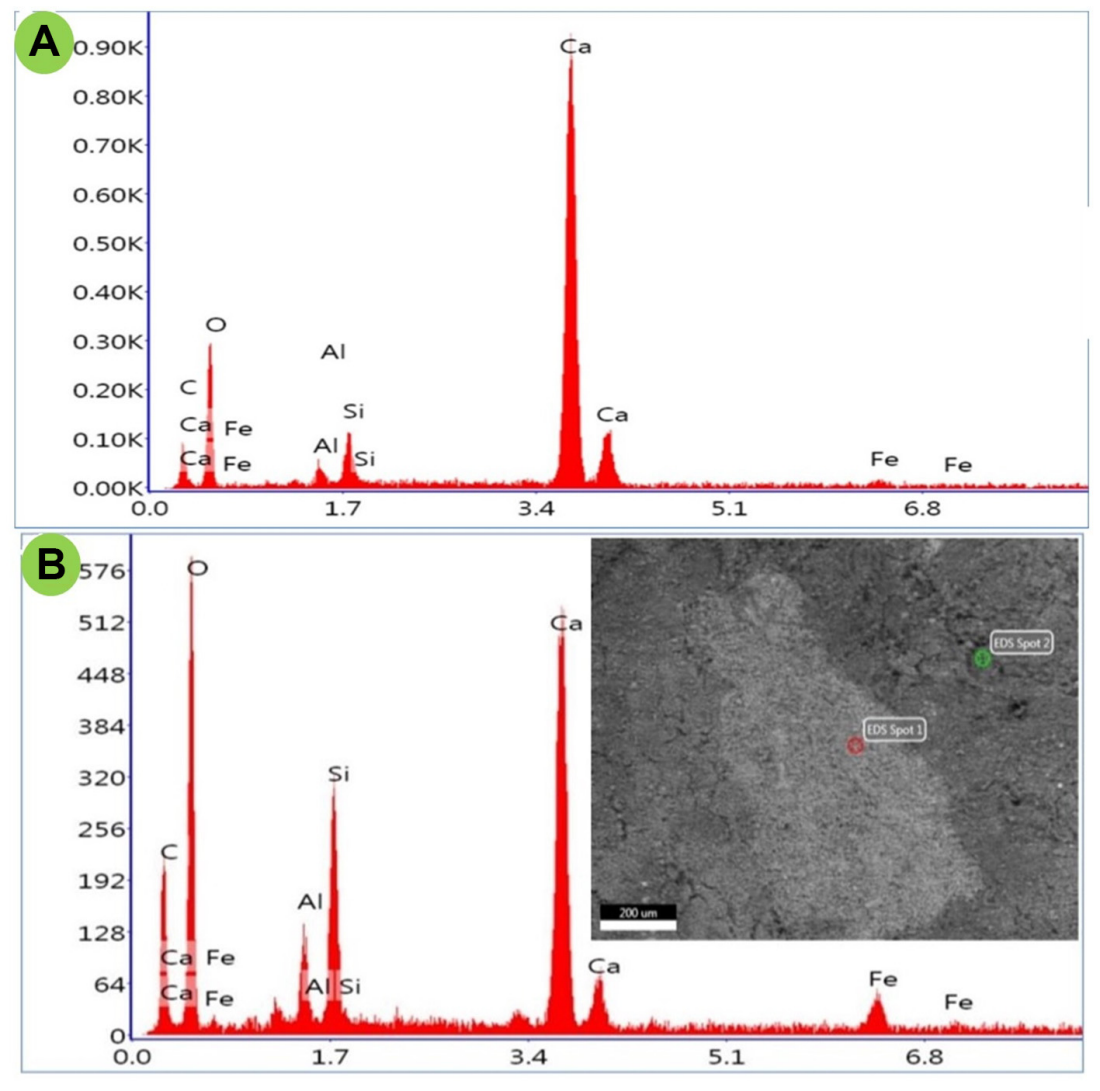
EDXS microanalysis of the stone elemental content; (A) control, (B) treatment (deteriorated) cubes.

The changes in the chemical properties of the artificially deteriorated cubes compared to those of the control were investigated using ESEM/EDXS for stone materials, while paper, textile, and wood materials were analyzed using FTIR. In stone (carbonate) cubes, the microanalysis of EDXS showed that the calcium content decreased from 41.81 to 20.47%, the oxygen content increased from 49.07 to 53.94%, the aluminum content increased from 1.14 to 2.57%, the silicon content increased from 2.88 to 6.57%, and the iron content increased from 1.23 to 4.27%. In the infected sample, some changes were detected in the concentrations of elements, such as iron, which increased from 1.3 to 14.5%, indicating the production of stains by *A. fumigatus* NMEC-PSTW.1. The data are shown in Table S5 and Fig. 7.

Upon analyzing the papers on textiles and wood materials, it was found that the growth of microorganisms and the breaking of bonds between cellulose molecules were responsible for these changes. This indicates that the degradation of large organic compounds, with a specific chemical functional group, occurs when decomposing fungal spores secrete extracellular enzymes. As a result, these compounds are converted into smaller compounds with different chemical functional groups. The FTIR analyses (Fig. S5A–S5C) revealed a change in the shape of the (OH) peak, which indicates an increase in the number of alcohol (OH) groups in the wavenumber range (3,300-3,500 cm^-1^); a change in the shape of the (C-H) peak, which indicates an increase in the number of C-H groups in the wavenumber range (2,800-3,000 cm^-1^); a change in the shape of the (OH) peak, which indicates an increase in the number of OH groups in the wavenumber range (1,300-1,500 cm^-1^); and a change in the shape of the (C-H) peak, which indicates an increase in the number of C-H groups in the wavenumber range (1,350-1,480 cm^-1^). In addition, the shape of the (C-O) peak changed.

## Discussion

The preservation of ancient monuments is a crucial aspect of the conservation of cultural heritage [45]. Cultural heritage monuments are exposed to chemical and physical processes that can alter their structure and composition. Furthermore, microorganisms that are linked to these monuments have the potential to cause degradation to both organic and inorganic materials. Diverse microorganisms inhabit a wide range of archaeological monuments. Microbial biodeterioration processes result from complex interactions of these microorganisms with the monument's surface and/or interior material as well as environmental and chemical factors [46].

Research into the microbial biodeterioration of monuments has significantly increased in the second half of the twentieth century. Biologically, monuments and their storage conditions can provide an ideal environment for microbial growth. These conditions exhibit substantial variations in physical factors like temperature, aeration, light, and the chemical nature of the substratum, nutrients, and water availability [28, 47].

Many historical sites in Egypt, such as pyramids, temples, tombs, and museums, are at risk of deterioration due to various microbial factors, including fungal infestation. Nevertheless, the persistent fungal infestation poses a significant threat to these iconic landmarks' structural integrity and longevity, resulting in their loss. Due to Egyptian monuments' historical and economic significance, substantial efforts are currently being made to protect them and avert their potential degradation [48]. As part of these efforts, the present study was conducted on ancient monuments stored at the NMEC in Cairo, Egypt. The museum, considered one of Egypt and the Arab world's largest museums, contains a multitude of archaeological artifacts composed of chemically different elements, including cellulose and carbonate materials.

Microorganisms of various species possess a wide range of metabolic and enzymatic versatility, making them suitable for numerous biotechnological applications [49-61]. Other types of microorganisms use their metabolic activities to degrade or decompose useful materials. Microorganisms, particularly fungi, have a significant impact on the biodeterioration of objects of cultural and historical significance. However, their detailed biochemical and eco-physiological functions and roles remain unclear [47]. In our study, 69 deteriorating fungal isolates were recovered from 22 archaeological objects examined at the NMEC in Cairo, Egypt. These objects are composed of four materials: paper, textiles, wood, and stones. Three of these objects consist of cellulose, specifically paper, textiles, and wood, whereas one is composed of stone.

According to the number of colonizing fungi, these archaeological objects were categorized as stone (37.68%), followed by paper (27.53%) and textiles (20.28%). Wooden objects (14.49%) contained the fewest fungal isolates. A relatively high correlation was detected between the number of fungal isolates found and the number of samples taken for all objects examined, except for paper objects where the number of fungal isolates was relatively higher than the expected number. These findings demonstrate that stone has the highest incidence of fungal deteriogens, the outcome of which requires more attention due to the significance of stone in art and architecture. Winkler [62] reported that limestone is one of the most common materials in historic structures. Additionally, ElBaghdady *et al*. [8] reported that limestone symbolizes the cultural legacy of humanity throughout history.

We attribute the high fungal infestation of the stone to the high number of archaeological stone objects examined compared to the other objects examined in this study. Although fungi are known to be heterotrophic microorganisms, most of the fungal isolates recovered in this study were from stone objects mainly composed of inorganic (carbonate) material. This finding can be due to fungi being well adapted to nutrient-poor conditions. Moreover, using finishing paints on these materials promotes fungal deterioration. Our findings are consistent with those of Suihko *et al*. [63], who reported that most stone-inhabiting heterotrophic fungi have deficient nutrient requirements.

Although many previous studies have investigated the biodeterioration of ancient Egyptian monuments at various Egyptian sites [7, 8, 40, 64, 65], most have focused on limestone archaeology. Therefore, our study is the first to address the different types of archaeological objects, including paper, textiles, wood, and stone. To our knowledge, there are few reports on the deterioration of various archaeological objects, such as wood, bone, limestone, baskets, and pottery, caused by biogenic fungi [23]. However, there are no reports on the archaeological objects in the NMEC.

The isolation and identification of various fungal species in museums, archives, and libraries are crucial not only for understanding their involvement in biogenic deterioration but also due to their significant impact on the health of workers and visitors. Ferrándiz-Pulido *et al*. [66] reported that various fungi colonizing historical monuments could be considered sources of human diseases such as respiratory diseases, allergies, skin infections, and phaeohyphomycosis. Several fungal genera, including *Acremonium*, *Alternaria*, *Arthobotrys*, *Aspergillus*, *Auerobasidium*, *Cladosporium*, *Curvularia*, *Drechslera*, *Fusarium*, and *Helminthosporium*, have been isolated from different monumental materials in various countries [67, 68]. Based on polyphasic identification of macroscopic and microscopic characteristics [69, 70], the fungal isolates obtained were found to belong to three genera: *Aspergillus* spp. (75.0%), *Penicillium* spp. (16.66%) and *Trichoderma* sp. (8.33%).

The accurate identification of fungi is crucial for various biological goals, including assessing biodiversity and biological activity, taxonomy, and species conservation [71]. Conventional identification methods based on fungal morphology and other phenotypic characteristics are time-consuming. Additionally, they require a certain level of morphological and taxonomic expertise. To overcome these limitations, DNA barcoding targeting numerous genetic loci, such as ITS regions, has been evaluated in fungi [72]. In the present study, molecular identification techniques, in particular amplification and sequencing of ITS genes and appropriate regions of the β-tub and TEF-1-α genes, were used to confirm the traditional (morphological) identification of these fungal species. The results showed a high degree of similarity with related species in the NCBI database, ranging from 99.33% to 100.0%.

These findings are consistent with a prior study conducted by Mohamed and Ibrahim [64], which revealed that *Aspergillus* was the predominant genus contaminating ancient limestone at different sites in Egypt, accounting for 46.6% of the total isolates obtained. Our results are also in agreement with those of Abdelhafez *et al*. [40], who reported that the genus *Aspergillus* spp., followed by *Penicillium* spp., was the predominant species isolated from deteriorated marble from three sites in Cairo, Egypt.

Out of the nine fungal species that were identified, *A. fumigatus* was found to be one of the most predominant species with the highest number of isolates, accounting for 20.28% (*n* = 14) of the total isolates (*n* = 69), followed by *A. flavus*, *A. parvisclerotigenus*, and *A. ardalensis* with 15.94% (*n* = 11), 11.59% (*n* = 8) and 8.69% (*n* = 6), respectively. Other fungal species also exhibited variations in their occurrence but with a lower number of isolates.

The study of the frequency of the identified species in the studied archaeological objects also indicated that *A. fumigatus* was categorized as a common frequency class (with a frequency of 100%), along with three other *Aspergillus* spp. Although the number and type of fungal species identified may vary depending on the geographical location, the type of archaeological objects examined, and the environmental conditions in which they are found in each study. Many previous local and international studies have reported the prevalence of *A. fumigatus* among other fungal species studied for its ability to cause biogenic deterioration of different types of ancient archaeological objects.

Consistent with our findings, Gupta [39] reported the identification of 13 fungal species associated with the degradation and deterioration of monuments, namely, Mahadev and Surya temples, in Narayanpur, India. Among them, *A. fumigatus* isolates were common (with a high percentage frequency of 100%) in all the samples examined. Additionally, Farooq *et al*. [73] identified 19 fungal species belonging to thirteen genera, causing mycobial deterioration in stone monuments in Dharmarajika, Taxila, Pakistan. *A. fumigatus* was the predominant species among the detected *Aspergillus* ssp. and the second most prevalent species among all identified species.

Furthermore, Abdel-Azeem *et al*. [74] reported similar results in a local study, where they observed that *A. fumigatus* was the second most prevalent species among nine different fungal species involved in the degradation of old archaeological wood from the Middle Cemetery of Abydos, Egypt. Relatively similar results were reported by Mohamed and Ibrahim [64]. They found that *A. niger* and *A. terreus* were the most common and dominant fungal species, respectively, while *A. fumigatus* was found in 71.4% of the archaeological sites. The disparity between our study and similar studies can be ascribed to the distinct geographical areas examined in each study. To our knowledge, there are no reports on the deterioration of the same archaeological objects to compare our results.

One of our main objectives in the current study was to identify an effective natural inhibitor to protect monuments from fungal attacks. Accordingly, the present study was extended to assess the efficacy of some synthetic microcides and natural products against isolated fungi. The antifungal activities of five synthetic microcides (CB, DCX, PCMC, SA, and TEAB) and three natural essential oils (clove, peppermint, and thyme) against the deteriorating fungi were assessed.

The antifungal activity results demonstrated that certain synthetic microcides exhibited a strong ability to inhibit the growth of all treated fungal species. These microcides had varying minimum inhibitory concentration (MIC) values: SA (100 ppm), CB (100-200 ppm), TEAB (400 ppm), and DCX (800 ppm). However, PCMC was ineffective in inhibiting the growth of all the tested fungi at all the tested concentrations (100-1,000 ppm). These results demonstrate that SA has the lowest MIC and is the most effective antifungal agent against fungal pathogens. Similar results were obtained by Abdelhafez *et al*. [40], who concluded that 100 ppm sodium azide was the most effective treatment for inhibiting the growth of all microbial isolates at a minimal concentration.

Kelley and Rodriguez-Kaban [75] reported that sodium azide has a wide range of biological activities and has garnered attention for its potential applications in different fields as an antifungal agent. Xuehong and Jie [76] reported that both CB and TEAB are considered the most common antimicrobial quaternary ammonium compounds. They attack the cytoplasmic membrane and cause protein denaturation, leading to leakage of intracellular components and death of the microorganism. Previous studies have discussed the use of microcides to inhibit the growth of degrading microorganisms, *e.g.*, the use of DCX by Ammar and El-Deeb [77], DCX and SA by Abdelhafez *et al*. [40], CB, PCMC, and TEAB by both Mohamed and Ibrahim [64] and ElBaghdady *et al*. [8].

While synthetic fungicides are frequently utilized to inhibit the growth of deteriorating fungi for the conservation of different archaeological monuments, they are not environmentally friendly for many indoor applications. Therefore, it is necessary to seek naturally existing alternatives that are both eco-friendly and pose minimal risk to human health [78].

The use of traditional biocidal products at cultural heritage sites has slowed due to the potential hazards they pose to human health and the environment. Therefore, many studies have been carried out to find innovative and environmentally friendly alternatives [79]. Natural extracts have demonstrated potential in protecting monuments against fungi. We examined the antifungal properties of three essential oils as part of an ongoing investigation into the use of natural products as bioagents to protect monuments against molds. Essential oils, which contain various fragrant and volatile compounds, have natural components such as monoterpenes, diterpenes, and hydrocarbons with different functional groups. These natural components contribute to the antibacterial and antifungal effects of essential oils [80].

The study results demonstrated that the tested oils (clove, peppermint, and thyme) exhibited potent antifungal activity by effectively inhibiting the growth of all treated fungal species. The MIC values were relatively low, with thyme oil having the lowest values (625-312 ppm), followed by peppermint and clove oil (1,250-2,500 ppm). These findings are consistent with previous studies conducted by Mironescu *et al*. [81], Preeti and Jain [82] and Mohamed and Ibrahim [64].

Essential oils such as thyme, cinnamon, peppermint, and clove have always been used as preservatives because they have been shown to have a robust microbicidal effect against a variety of microbial pathogens [83] by destabilizing the cytoplasmic membrane and acting as proton exchangers [84]. Some studies [85, 86] suggest that the compounds penetrate the cell, interfering with cell metabolism. In addition, phenols such as carvacrol and eugenol (the main component of the essential oil) disrupt the cell membrane and react with the active sites of essential enzymes.

Based on the aforementioned antifungal results, both thyme oil and sodium azide were found to be the most active growth inhibitors against all treated fungi. The MICs were 625 and 100 ppm, with ZI diameters of 19.0 ± 0.70 – 23.76 ± 1.15 and 39 13.30 ± 0.35 – 19.66 ± 0.54 mm, respectively. While the fungicides examined, particularly sodium azide, demonstrated lower MICs and larger ZI diameters than the tested essential oils, they are associated with numerous drawbacks. These include causing undesirable permanent alterations to archaeological objects and posing toxicity risks to humans and the environment [87]. In contrast, this study selected essential oils that demonstrated antifungal effects as control agents. Accordingly, the present study recommends the use of thyme oil as a natural inhibitor treatment to control the decay of the studied monuments.

The antifungal activity of thyme oil (*Thymus vulgaris*) against various types of fungal infections, including *Aspergillus* spp., has been well documented [88]. Thyme oil was used in ancient Egypt as an antiseptic for respiratory tract infections. Thyme oil contains 20–50% of the antiseptic compound thymol in addition to various other compounds, such as myrcene and p-cymene [89].

The present study did not investigate the potential damage caused by the solvents EA and DMSO on the structure of archaeological materials and decorative paints. Nevertheless, our hypotheses and knowledge are based on other previous studies reported by ElBaghdady *et al*. [8]. Researchers have investigated this point and used similar antimicrobial compounds (natural essential oils and synthetic chemical microcides) dissolved in ethyl alcohol to treat different microbial species. These compounds were used to treat the deterioration of some outdoor archaeological (stone) objects. They reported that no visual or chemical changes occurred in the materials of the archaeological objects examined. Moreover, another study by Osman *et al*. [90] reported that the application of DMSO in the treatment of deteriorating fungi did not affect the color, physical, or chemical properties of treated archaeological (wooden) objects. However, due to the various archaeological objects examined in this study, further experiments should be conducted to establish a protocol for the application of thyme oil (as a recommended natural treatment agent) and its dissolving solvent (DMSO, 5%) and to evaluate their interactions with different archaeological materials to ensure their safety.

One of the most important aspects of restoring and protecting cultural heritage objects is the early detection of material deterioration caused by microbial colonization. To understand the changes that occurred in the monuments due to fungal deterioration, we carried out a simulation of the deterioration process for model cubes made of paper, textiles, and wood deteriorated with a spore suspension of *A. fumigatus* NMEC–PSTW.1 For the stone material, visual observation of deterioration signs indicated pigmentation and gray decay spots. In addition, ESEM-EDAX examination and mechanical characterization of limestone cubes before and after deterioration revealed extensive colonization of the stone with fungal hyphae. This study agrees with that of Gadd [91] and Priester *et al*. [92], who reported that the penetration of fungal hyphae along the crystal plane by some fungi destabilizes the stone structure, leading to its mechanical deterioration.

Furthermore, elemental analysis (EDXS) of the inoculated stone cubes revealed a decrease in the calcium ratio and an increase in the aluminum, iron, oxygen, and silicon ratios, which are indicators of biodeterioration. These findings are consistent with those of Videla *et al*. [93]. The organic layer and metabolic products, such as organic acids or extracellular polymeric substances, are anticipated to contain significant amounts of carbon and oxygen. The calcium percentage was also found to be low, indicating that the limestone substrate was hidden beneath the organic biofilm layer [94].

Limestone undergoes a complex process known as deterioration, which alters its mechanical characteristics and mineralogical composition. When gypsum or a crust forms in the pores of limestone, it acts as a pore-sealing cement [95] and thus contributes to the formation of a nonporous surface crust on the limestone. This reduces the porosity of the stone and causes a weakening of the host rock [96]. This finding explains the compressive strength and porosity calculations determined in this study.

The current study has some limitations that should be addressed in future research. For example it is recommended that the number of natural products tested be increased and that products from different sources be included to broaden the applicability of treatment with eco-friendly products.

This study recorded the biogenic fungal deterioration of carbonate and cellulosic ancient Egyptian monuments at the NMEC in Cairo, Egypt. Detyriogenic fungi were identified as different species of *Aspergillus*, *Penicillium*, and *Trichoderma*. The results revealed that *A. fumigatus* was the most prevalent species among them. The natural essential oils clove, peppermint, and thyme, as well as the synthetic antimicrobial agents CB, DCX, SA, and TEAB, were able to inhibit the growth of the identified fungal species. Thyme oil and sodium azide demonstrated the highest effectiveness in inhibiting fungal growth. Essential oils are suitable for application on archaeological objects due to their effectiveness, safety for the environment, workers, and visitors, availability, low price, and lack of residue. In conclusion, this study recommends using thyme oil to prevent the deterioration of carbonate and cellulosic ancient monuments in the NMEC.

## Supplemental Materials

Supplementary data for this paper are available on-line only at http://jmb.or.kr.



## Figures and Tables

**Table 1:** ITS sequence identification of the deteriorating fungal species recovered from the examined archaeological objects.

Fungal species code	GenBank accession number	Homolog sequence	Sequence identity %	Closest accession number
NMEC–PSTW.1	PP562369	*Aspergillus fumigatus*	98.89%	KF003432
NMEC–PSTW.2	PP814820	*Aspergillus flavus*	99.32%	KX852295
NMEC–PSTW.3	PP814821	*Aspergillus oryzae*	99.09%	MN545453
NMEC–PSTW.4	PP814822	*Aspergillus flavipes*	99.31%	KP987083
NMEC–PTW.5	PP814823	*Aspergillus japonicus*	99.26%	KT151588
NMEC–PTW.6	PP814824	*Aspergillus parasiticus*	98.82%	ON797663
NMEC–ST.7	PP814825	*Aspergillus terreus*	98.73%	MT436785
NMEC–SW.8	PP814826	*Aspergillus aureus*	99.68%	LC573590
NMEC-PS.9	PP814827	*Aspergillus unguis*	99.46%	EF652497
NMEC–PSTW.10	PP814828	*Penicillium simplicissimum*	99.81%	MK584640
NMEC–P.11	PP814829	*Penicillium canescens*	98.38%	MK576131
NMEC–PT.12	PP814830	*Trichoderma* sp.	99.26%	OR710780

**Table 2:** BLAST analysis of β-tub and TEF-1 α gene and its accession number.

Fungal species code	GenBank accession number	Homolog sequence	Sequence identity %	Closest accession number
NMEC–PSTW.1	PQ212593	*Aspergillus fumigatus*	100.0%	MT196114.1
NMEC–PSTW.2	PQ212594	*Aspergillus flavus*	100.0%	MK942057.1
NMEC–PSTW.3	PQ212595	*Aspergillus parvisclerotigenus*	99.54%	JX627691.1
NMEC–PSTW.4	PQ212596	*Aspergillus ardalensis*	100.0%	LT798967.1
NMEC–PTW.5	PQ212597	*Aspergillus japonicus*	99.49%	AY820017.1
NMEC–PTW.6	PQ212598	*Aspergillus krugeri*	99.80%	MK450928.1
NMEC–ST.7	PQ212599	*Aspergillus terreus*	99.33%	JQ897354.1
NMEC–SW.8	PQ212600	*Aspergillus piperis*	100.0%	KJ469441.1
NMEC-PS.9	PQ212601	*Aspergillus pluriseminatus*	99.77%	KU866806.1
NMEC–PSTW.10	PQ212602	*Penicillium piscarium*	100.0%	DQ486649.1
NMEC–P.11	PQ212603	*Penicillium simplicissimum*	99.83%	DQ834935.1
NMEC–PT.12	PQ212604	*Trichoderma asperellum*	100.0%	MN428412.1

**Table 3:** Table 3

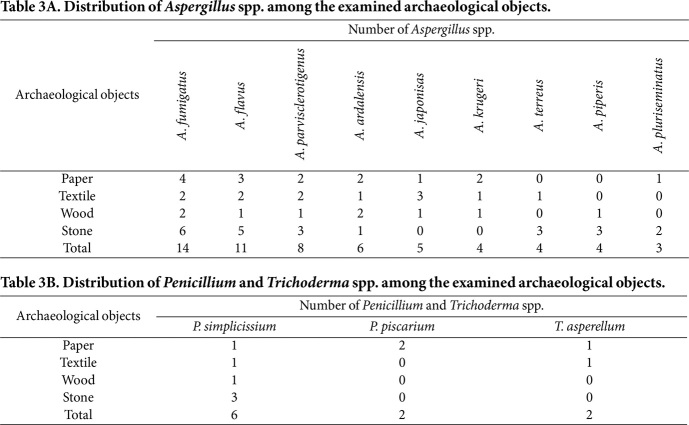

**Table 4:** Occurrence, percentage frequency and frequency class of the different fungal species among the examined archaeological objects.

Fungal species	Archaeological objects				Number of isolates	Frequency (%)	Frequency Class
	Paper	Textile	Wood	Stone			
*A. fumigatus*	+	+	+	+	14	100	C
*A. flavus*	+	+	+	+	11	100	C
*A. parvisclerotigenus*	+	+	+	+	8	100	C
*A. ardalensis*	+	+	+	+	6	100	C
*P. simplicissium*	+	+	+	+	6	100	C
*A. japonicus*	+	+	+	–	5	75	F
*A. krugeri*	+	+	+	–	4	75	F
*A. piperis*	–	–	+	+	4	50	O
*A. terreus*	–	+	–	+	4	50	O
*A. pluriseminatus*	+	–	–	+	3	50	O
*P. piscarium*	+	–	–	–	2	25	R
*T. asperellum*	+	+	–	–	2	50	O

(+) = presence of species; (-) = absence of species; C = common; O = Occasional; F = frequent, R = rare

**Table 5:** Table 5

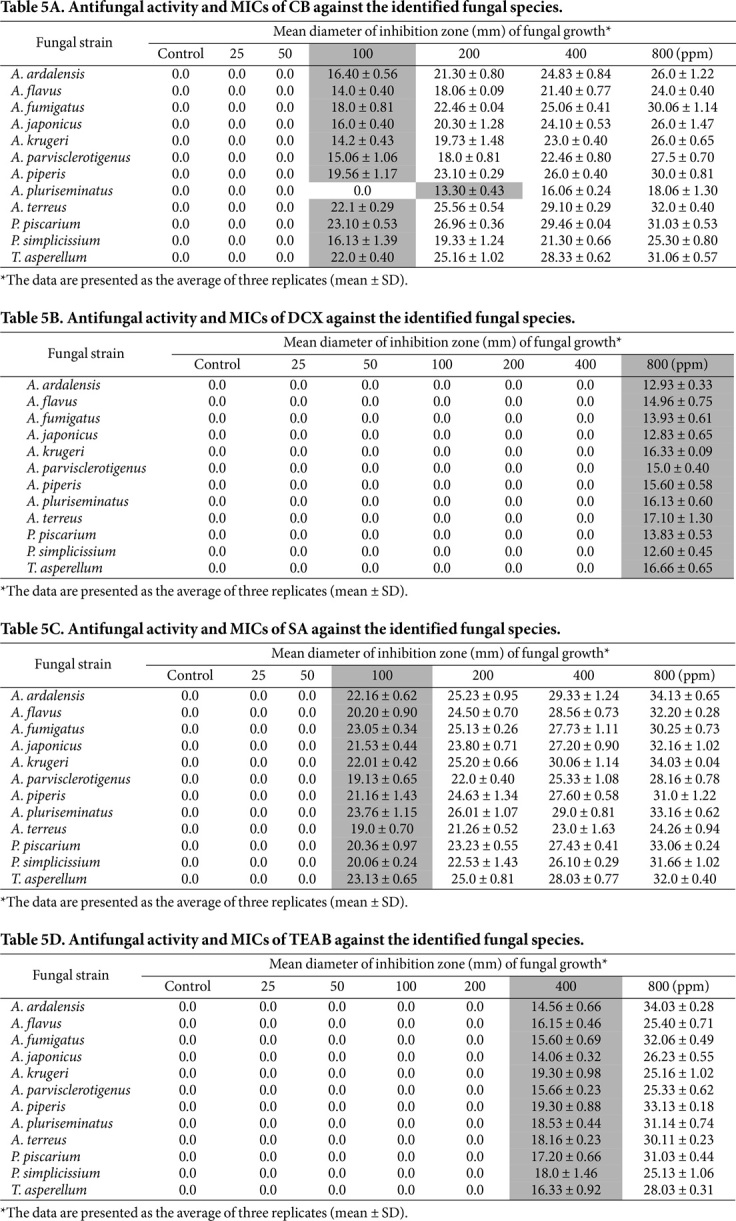

**Table 6:** Table 6