# Supplementary material for: Natural Inhibitory Treatment of Fungi-Induced Deterioration of Carbonate and Cellulosic Ancient Monuments: Isolation, Identification and Simulation of Biogenic Deterioration
Source: J Microbiol Biotechnol. 2024 Aug 30;34(10):2049–69. doi: 10.4014/jmb.2404.04032 (PMC11540613; doi:10.4014/jmb.2404.04032)
Supplement: Supplementary file 1 [file jmb-34-10-2049-supple.pdf]

Table S1. The selected deteriorated archaeological objects; their types, descriptions and collected samples for each.

No.	Archaeological objects	Objects description	Number of samples	Type of sampling
1	Paper (n = 2)	Holy Quran (PA)	4	4 Swabbing
2		Holy Quran (PB)	4	4 Swabbing
3	Textile (n = 5)	Flax around mummy (Enshroud) (TA)	3	3 Swabbing
4		Flax fabric by fees (TB)	2	2 Swabbing
5		Wool around mummy (Enshroud) (TC)	3	3 Swabbing
6		Wool around mummy (Enshroud) (TD)	2	2 Swabbing
7		Wool around mummy (Enshroud) (TE)	1	1 Swabbing
8	Wooden (n = 5)	Bed from wood and hull rice (WA)	2	2 Swabbing
9		Coffin with color painting (WB)	2	1 Swabbing and 1 needle
10		Coffin with color painting (WC)	2	1 Swabbing and 1 needle
11		Small armoire (Naws) (WD)	1	1 Swabbing
12		Statute with color painting (WE)	1	1 Needle
13	Stone (n = 10)	Bowl from limestone B1 (SA)	5	2 Swabbing and 3 scratching
14		Bowl from limestone B2 (SB)	4	1 Swabbing and 3 scratching
15		Canobic jar from limestone (SC)	2	1 Swabbing and 1 needle
16		Grindstone from chert (SD)	7	1 Swabbing, 3 needle and 3 scratching
17		Small flask to storied perfume (SE)	1	1 Swabbing
18		Statue from limestone (SF)	2	1 Swabbing and 1 scratching
19		Statute with color painting (SG)	2	1 Swabbing and 1 scratching
20		Stele with paintings and color (SH)	1	1 Swabbing
21		Stele with paintings and color (SI)	1	1 Swabbing
22		Vessel from limestone (SJ)	6	2 Swabbing, 3 needle and 1 scratching
**Total**	**22 Types**	**Total**	**58 Samples**	36 swabs; 10 needles and 12 scratches

*Each type of archeological monument sampled was coded with the following letters: paper (PA-PB), textiles (TA-TE), wood (WA-WE) and stone (SA-SJ).

Table S2. The deteriorating fungal isolates recovered from the archaeological objects and their growth characteristics.

Archaeological objects	Isolate code	Isolation medium	Colony morphology			
			Shape	Color	Pigmentation	Growth
Papers (19)	NMEC –P1	CZDA, PDA, MEA	Filamentous	Bluish green	ND	+++
	NMEC–P2	CZDA	Irregular	Blackish purple	ND	+++
	NMEC–P3	CZDA	Circular	Bluish green	ND	+++
	NMEC–P4	CZDA	Filamentous	Yellowish white	ND	++
	NMEC–P5	CZDA	Filamentous	Grayish green	ND	+
	NMEC–P6	CZDA	Rhizoid	Buff to brown	ND	+
	NMEC–P7	CZDA	Irregular	Deep green	ND	+++
	NMEC–P8	CZDA	Filamentous	Olive buff	ND	+++
	NMEC–P9	CZDA	Filamentous	Yellowish brown	ND	+
	NMEC–P10	CZDA	Circular	Pale yellow	ND	+
	NMEC–P11	All used media	Circular	Buff to yellow	Deep brown	+
	NMEC–P12	CZDA	Rhizoid	Blackish brown	ND	+++
	NMEC–P13	CZDA	Irregular	Whitish green	ND	+++
	NMEC–P14	CZDA	Irregular	Yellowish green	ND	++
	NMEC–P15	CZDA	Rhizoid	Greenish yellow	Light brown	+++
	NMEC–P16	CZDA	Filamentous	Light brown	ND	++
	NMEC–P17	CZDA	Circular	Yellowish brown	ND	+
	NMEC–P18	CZDA	Filamentous	Deep green	ND	+++
	NMEC–P19	CZDA	Filamentous	Yellowish green	ND	++
Textiles (14)	NMEC–T20	PDA	Irregular	Yellowish buff	ND	++
	NMEC–T21	PDA	Rhizoid	Olive buff	Light brown	+++
	NMEC–T22	PDA	Filamentous	Yellowish green	ND	++
	NMEC–T23	PDA	Filamentous	Grayish green	ND	+
	NMEC–T24	PDA	Irregular	Light brown	ND	++
	NMEC–T25	PDA	Irregular	Grayish white	ND	+
	NMEC–T26	PDA	Rhizoid	Blackish purple	ND	+++
	NMEC–T27	PDA	Rhizoid	Bluish green	ND	+++
	NMEC–T28	PDA	Filamentous	Light green	ND	+++
	NMEC–T29	PDA	Irregular	Light yellow	ND	++
	NMEC–T30	PDA	Irregular	Whitish green	ND	+++
	NMEC–T31	PDA	Filamentous	Yellowish green	ND	++
	NMEC–T32	PDA	Filamentous	Deep brown	ND	+++
	NMEC–T33	PDA	Rhizoid	Greenish yellow	ND	+++
Wooden (10)	NMEC–W34	PDA	Rhizoid	Deep green	ND	+++
	NMEC–W35	PDA	Irregular	Whitish green	ND	+++
	NMEC–W36	PDA	Irregular	Greenish yellow	ND	++

	NMEC–W37	PDA	Rhizoid	Deep green	ND	+++
	NMEC–W38	PDA	Rhizoid	Olive buff	ND	+++
	NMEC–W39	PDA	Filamentous	Yellowish green	ND	++
	NMEC–W40	PDA	Filamentous	Yellowish brown	ND	+++
	NMEC–W41	PDA	Filamentous	Blackish brown	ND	+++
	NMEC–W42	PDA	Irregular	Bluish green	ND	+++
	NMEC–W43	PDA	Irregular	Yellowish green	ND	++
Stone (26)	NMEC–S44	CZDA	Rhizoid	Greenish yellow	ND	+++
	NMEC–S45	CZDA	Irregular	Grayish white	ND	+
	NMEC–S46	CZDA	Filamentous	Whitish green	ND	+++
	NMEC–S47	CZDA	Circular	Yellowish brown	ND	+
	NMEC–S48	CZDA	Rhizoid	Buff to brown	ND	+
	NMEC–S49	CZDA	Filamentous	Deep green	ND	+++
	NMEC–S50	CZDA	Circular	Brown	Light brown	+
	NMEC–S51	CZDA	Irregular	Deep green	ND	+++
	NMEC–S52	CZDA	Rhizoid	Brownish buff	ND	+
	NMEC–S53	CZDA	Filamentous	Pale yellow	ND	+
	NMEC–S54	CZDA	Irregular	Deep green	ND	+
	NMEC–S55	CZDA	Rhizoid	Blackish brown	ND	+++
	NMEC–S56	CZDA	Circular	Light brown	Deep brown	+
	NMEC–S57	CZDA	Irregular	Yellowish buff	ND	++
	NMEC–S58	CZDA	Filamentous	Yellowish green	ND	++
	NMEC–S59	CZDA	Filamentous	Light brown	ND	++
	NMEC–S60	CZDA	Irregular	Deep brown	ND	+++
	NMEC–S61	CZDA	Rhizoid	Bluish green	ND	+++
	NMEC–S62	CZDA	Filamentous	Yellowish green	ND	++
	NMEC–S63	CZDA	Irregular	Grayish white	ND	+
	NMEC–S64	CZDA	Filamentous	Light yellow	ND	++
	NMEC–S65	CZDA	Circular	light brown	brown	+
	NMEC–S66	CZDA	Rhizoid	Buff to brown	ND	+
	NMEC–S67	CZDA	Filamentous	Deep green	ND	+++
	NMEC–S68	CZDA	Rhizoid	Blackish purple	ND	+++
	NMEC–S69	CZDA	Irregular	Light yellow	ND	++

CZDA (Czapek–Dox agar), PDA (potato dextrose agar). The following growth degree symbols were used: ND (not detected), - (negative), + (good), ++ (moderate), and +++ (abundant).

Table S3-A. Morphological characteristics of identified *Aspergillus fumigatus* isolates (NMEC-P1, P4, P9, P18, T25, T27, W40, W42, S49, S51, S54, S61, S64 and S67).

Character	Examination results
* Culture examination:	
Growth characteristics	Colonies reaching 2–3 cm diameter in 7 days at 28°C, on Czapek medium, blue green colonies, reverse colorless
* Microscopic examination:	
1. Conidial heads	Short columnar
2. Conidiophore	12.0 µm in diameter
3. Vesicle	Flasked shape 23.0 µm
4. First Sterigmata	7.9 X 3.0 µm
5. Conidia	Conidia, subspherical 3.0 µm

Table S3-B. Morphological characteristics of identified *Aspergillus flavus* isolates (NMEC-P3, P6, P10, T22, T23, W38, S57, S58, S60, S62 & S68).

Character	Examination results
* Culture examination:	
Growth characteristics	Colonies fast-growing, reaching 5-7 cm diameters in four days at 25°C on malt media; usually consist of a dense felt of yellowish green mycelia, with pale yellow reverse
* Microscopic examination:	
1. Conidiophores	Conidiophores are coarsely roughened, greenish yellow in color, 9.5 µm in diameter
2. Vesicles	Subglobose, 16.5 µm in diameter
3. Sterigmata	Sterigmata in one series, 7.0 – 3.4 µm
4. Conidia	Conidia globose, 4.0 µm in diam. Yellow–green conspicuously rough-walled

Table S3-C. Morphological characteristics of identified *Aspergillus oryzae* strains (NMEC-P5, P12, T24, T32, W39, S44, S46 & S55).

Character	Examination results
* Culture examination:	
Growth characteristics	Colonies reaching 5-6 cm diameter in 7 days at 28°C, on Czapek, greenish yellow to olive buff, reverse brown with age
* Microscopic examination:	
1. Conidial heads	Radiate
2. Conidiophore	12.0 µm in diameter
3. Vesicle	Subglobose 23.0 µm
4. First sterigmata	12 X 3.5 µm
5. Second sterigmata	9.0 X 3.0 µm
6. Conidia	Conidia, spherical 4.5 µm

Table S3-D. Morphological characteristics of identified *Aspergillus flavipes* isolates (NMEC-P7, P13, T33, W37, W41 and S47).

Character	Examination results
* Culture examination:	
Growth characteristics	Colonies on Czapek agar at 25 C attaining a diameter of 3.0-3.5 cm within 7 days gives buff to yellow brown. Reverse deep brown pigment observed
* Microscopic examination:	
1. Conidial heads	Columnar
2. Vesicle diameter	Subglobose 9.0 µm in diameter
3. Primary sterigmata	5.5X2.5 µm
4. Secondary sterigmata	4.0X2.0 µm.
5. Conidiophore diameter	3.0 µm in diameter.
6. Conidia	Globose, smooth, 2.0 µm in diameter

Table S3-E. Morphological characteristics of identified *Aspergillus japonisas* isolates (NMEC-P8, T20, T26, T31 and W36).

Character	Examination results
* Culture examination:	
Growth characteristics	Colonies reaching 5-7 cm diameter in 7 days at 28°C, on Czapek, velvety, black colonies, reverse first colorless then purple brown.
* Microscopic examination:	
1. Conidial heads	Black, radiate
2. Conidiophore	9.5 µm in diameter
3. Vesicle	Globose - subglobose, 28.0 µm
4. First sterigmata	Uniseriate, 7.7 X 4.5µm
5. Conidia	Conidia sub - globose, 3.5 µm

Table S3-F. Morphological characteristics of identified *Aspergillus parasiticus* isolates (NMEC-P11, P17, T28 and W43).

Character	Examination results
* Culture examination:	
Growth characteristics	Colonies fast-growing, reaching 5-7 cm diameters in four days at 25°C on malt media; usually consist of a dense felt of yellowish green mycelia, with pale yellow reverse
* Microscopic examination:	
1. Conidiophore	Conidiophores are coarsely roughened, geenish yellow in color, 10.4 µm in diameter
2. Vesicle	Subglobose, 24.5 µm in diameter
3. Sterigmata	Sterigmata in one series, 8.6 – 3..3 µm.
4. Conidia	Conidia globose, 3.5 µm in diam. Yellow–green, conspicuously rough-walled

Table S3-G. Morphological characteristics of identified *Aspergillus terreus* isolates (NMEC-T30, S48, S52 and S66).

Character	Examination results
* Culture examination:	
Growth characteristics	Colonies on Czapek agar at 25 □C attaining a diameter of 3.0-3.5 cm within 7 days gives buff to brown
* Microscopic examination:	
1. Conidial heads	Columnar, 60.5 µm
2. Vesicle diameter	Subglobose 15.4 µm in diameter
3. Primary sterigmata	6.4X2.3 µm
4. Secondary sterigmata	6.0X1.2 µm
5. Conidiophore diameter	5.2 µm in diameter.
6. Conidia	Globose, smooth, 2.1 µm in diameter

Table S3-H. Morphological characteristics of identified *Aspergillus aureus* isolates (NMEC-W35, S50, S56 and S65).

Character	Examination results
* Culture examination:	
Growth characteristics	Colonies on Czapek agar at 25 □C attaining a diameter of 3.0-3.5 cm within 7 days gives buff to yellow brown. Reverse deep brown pigment observed
* Microscopic examination:	
1. Conidial heads	Columnar
2. Vesicle diameter	Subglobose 12.0 µm in diameter.
3. Primary sterigmata	5.0X2.0 µm
4. Secondary sterigmata	4.2X1.6 µm
5. Conidiophore diameter	4.0 µm in diameter
6. Conidia	Globose, smooth, 2.0 µm in diameter

Table S3-I. Morphological characteristics of identified *Aspergillus unguis* isolates (NMEC-P15, S59 & S69).

Character	Examination results
* Culture examination:	
Growth characteristics	Colonies reaching 2-3 cm diameter in 7 days at 28°C, Light yellow to light brown colonies on Czapek, reverse brown in color
* Microscopic examination:	
1. Conidial heads	Radiate to loosely columnar
2. Conidiophore	12.0 µm in diameter
3. Vesicle	10.5 µm
4. First Sterigmata	7.0 X 3.0 µm
5. Second Sterigmata	6.0 X 2.4 µm
6. Conidia	Conidia, sub spherical 3.0 µm

Table S3-J. Morphological characteristics of identified *Penicillium simplicissium* isolates (NMEC-P16, T21, W34, S45, S53 & S63).

Character	Examination results
* Culture examination:	
Growth characteristics	Colonies on CYA attending 4-6 cm diameter at 25°C, white, grayish, buff, pale yellow mycelium to deep green. Reverse, pale, to yellowish brown. Micro colonies growth on CYA at 5°C and 37°C
* Microscopic examination:	
1. Penicillus type	Biverticillate
2. Conidiophore	Conidiophore diamerter 3.0 µm
3. Metulae	12.2X3.0 µm
4. Phialides	7.0 X 2.0 µm
5. Conidia	Conidia subspherical 3.5X 2.6 µm

Table S3-K. Morphological characteristics of identified *Penicillium canescens* isolates (NMEC-P4 and P19).

Character	Examination results
* Culture examination:	
Growth characteristics	Gray to deep green colonies on CYA with deep green reverse. Micro colonies at 5°C and 37°C
* Microscopic examination:	
1. Penicillus type	Biverticillate, sometimes terverticellate.
2. Rami	17.5X4.1 µm
3. Metulae	14.2X3.0 µm
4. Phialides	5.8X2.4 µm
5. Conidia	Conidia globose, smooth walled 3.0 µm

Table S3-L. Morphological characteristics of identified *Trichoderma harzianum* isolates (NMEC-P2 and T29).

Character	Examination results
* Culture examination:	
Growth characteristics	Colonies reaching 5-7 cm diameter in ten days at 28°C, on malt, whitish to green color
* Microscopic examination:	
1. Phialides	In whorls of 2–4 flask shaped, 4.0X2.5 µm
2. Conidia	Conidia spherical, green in mass 3.2X2.0 µm
3. Chlamydospores	Abundant terminal and intercalary

Table S4-A. MICs of synthetic microcides for each deteriorating fungal species and the resulting zone of inhibition diameter in mm.

Fungal strain	CB				SA		TEAB	
	ppm	ZI (mm)	ppm	ZI (mm)	ppm	ZI (mm)	ppm	ZI (mm)
A. aureus	100	19.56 ± 1.17	800	15.60 ± 0.58	100	21.16 ± 1.43	400	19.30 ± 0.88
A. flavipes	100	16.40 ± 0.56	800	12.93 ± 0.33	100	22.16 ± 0.62	400	14.56 ± 0.66
A. flavus	100	14.0 ± 0.40	800	14.96 ± 0.75	100	20.20 ± 0.90	400	16.15 ± 0.46
A. fumigatus	100	18.0 ± 0.81	800	13.93 ± 0.61	100	23.05 ± 0.34	400	15.60 ± 0.69
A. japonicus	100	16.0 ± 0.40	800	12.83 ± 0.65	100	21.53 ± 0.44	400	14.06 ± 0.32
A. oryzae	100	15.06 ± 1.06	800	15.0 ± 0.40	100	19.13 ± 0.65	400	15.66 ± 0.23
A. parasiticus	100	14.2 ± 0.43	800	16.33 ± 0.09	100	22.01 ± 0.42	400	19.30 ± 0.98
A. terreus	100	22.1 ± 0.29	800	17.10 ± 1.30	100	19.0 ± 0.70	400	18.16 ± 0.23
A. unguis	200	13.30 ± 0.43	800	16.13 ± 0.60	100	23.76 ± 1.15	400	18.53 ± 0.44
P. canescens	100	23.10 ± 0.53	800	13.83 ± 0.53	100	20.36 ± 0.97	400	17.20 ± 0.66
P. simplicissium	100	16.13 ± 1.39	800	12.60 ± 0.45	100	20.06 ± 0.24	400	18.0 ± 1.46
T. harzianum	100	22.0 ± 0.40	800	16.66 ± 0.65	100	23.13 ± 0.65	400	16.33 ± 0.92

Table S4-B. MICs of the essential oils for each deteriorating fungal species and the resulting zone of inhibition diameter in mm.

Fungal strain	Clove oil		Peppermint oil		Thyme oil	
	ppm	ZI (mm)	ppm	ZI (mm)	ppm	ZI
A. aureus	5.0	13.03 ± 0.44	2.50	13.10 ± 0.37	0.625	19.30 ± 0.88
A. flavipes	1.25	16.63 ± 1.34	1.25	16.01 ± 0.64	0.625	16.16 ± 0.38
A. flavus	2.50	15.0 ± 1.14	2.50	16.90 ± 0.37	0.625	16.96 ± 0.36
A. fumigatus	1.25	13.76 ± 0.61	1.25	13.60 ± 0.53	0.312	15.30 ± 0.49
A. japonicus	2.50	17.06 ± 0.24	2.50	14.83 ± 0.16	0.312	14.20 ± 0.16
A. oryzae	1.25	14.30 ± 1.36	1.25	15.36 ± 1.0	0.312	15.23 ± 0.26
A. parasiticus	2.50	17.08 ± 0.42	2.50	14.0 ± 0.26	0.625	13.30 ± 0.35
A. terreus	2.50	14.70 ± 0.57	2.50	13.33 ± 0.24	0.625	15.63 ± 0.57
A. unguis	2.50	15.0 ± 1.46	2.50	14.46 ± 0.73	0.312	12.43 ± 0.98
P. canescens	2.50	13.50 ± 0.81	2.50	12.90 ± 0.53	0.625	17.70 ± 0.21
P. simplicissium	5.0	13.60 ± 1.45	2.50	13.06 ± 0.24	0.312	14.03 ± 0.53
T. harzianum	1.25	13.96 ± 0.12	1.25	13.93 ± 0.32	0.625	17.20 ± 0.16

Table S5. EDXS analysis of the chemical changes in the stone cubes.

Element	Material weight (%)	
	Control	Treatment
Aluminum	1.14%	2.57%
Iron	1.23%	4.27%
Silicon	2.88%	6.57%
Carbon	3.87%	12.19%
Calcium	41.81%	20.47%
Oxygen	49.07%	53.94%

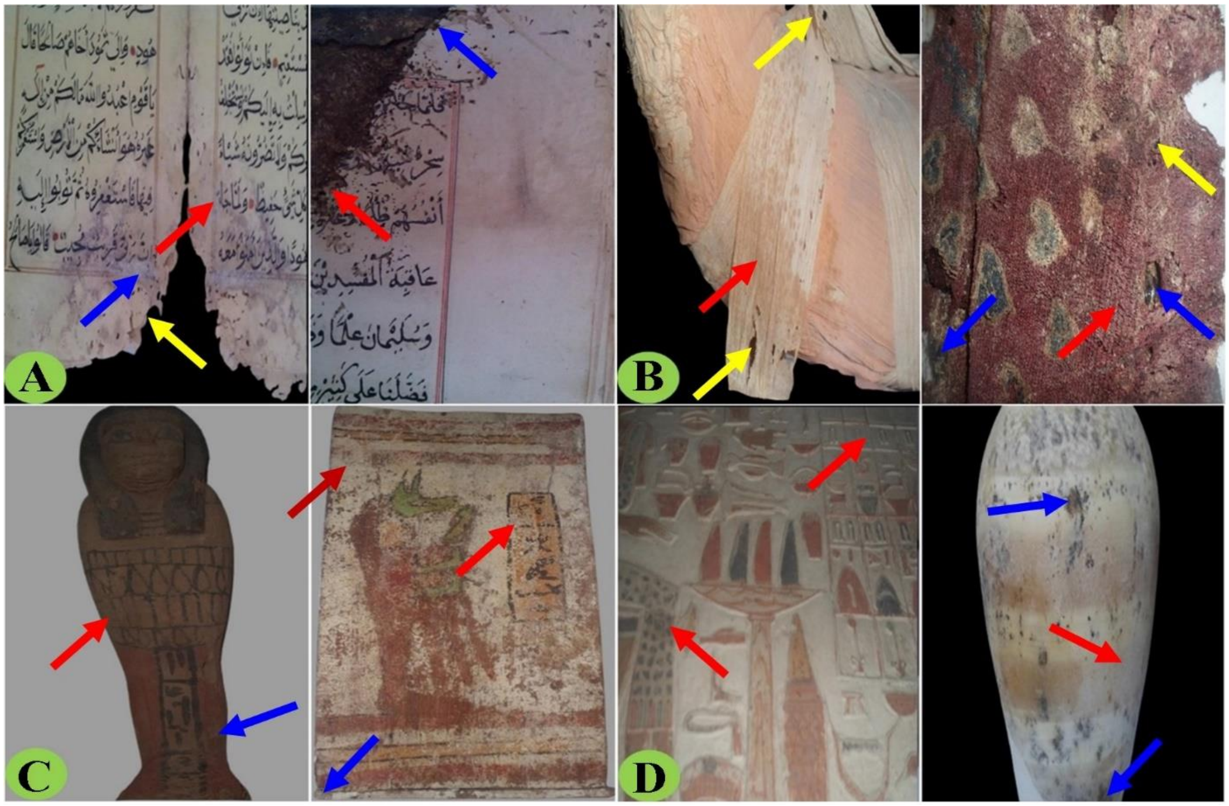

Fig. S1. The selected archaeological objects. (A) paper, (B) textile, (C) wood, and (D) stone. The signs of fungal deterioration are represented by red arrows for discoloration, blue arrows for decay, and yellow arrows for biopitting and cracking.

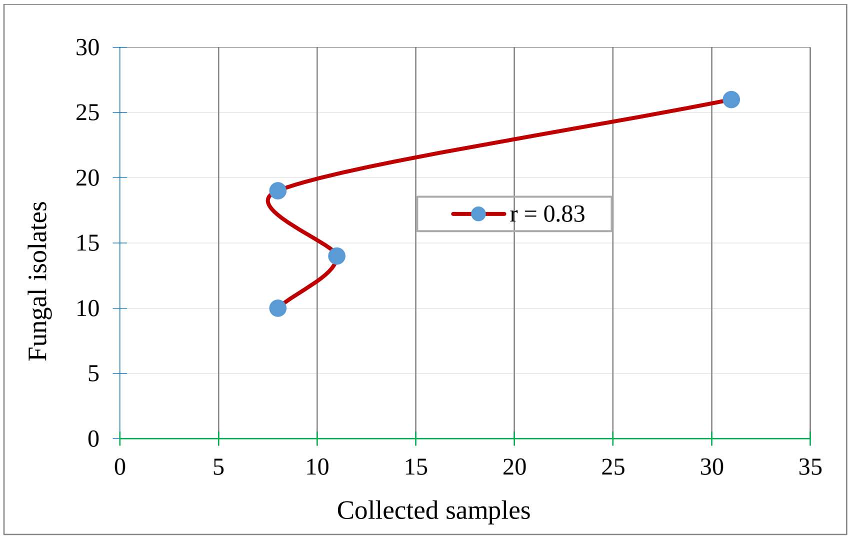

Fig. S2. The correlation between the number of fungal isolates obtained and the number of samples collected from the examined archaeological objects.

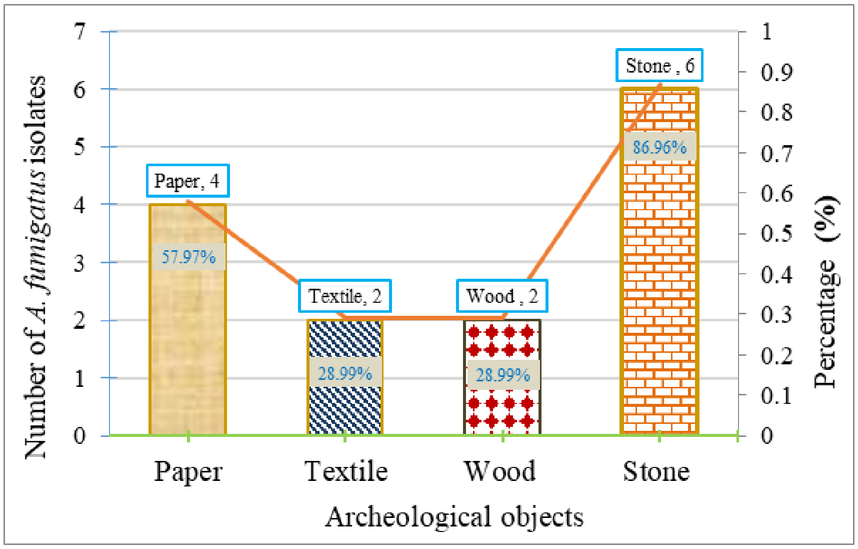

Fig. S3. Distribution of *A. fumigatus* NMEC–PSTW.1 among the examined archaeological objects.

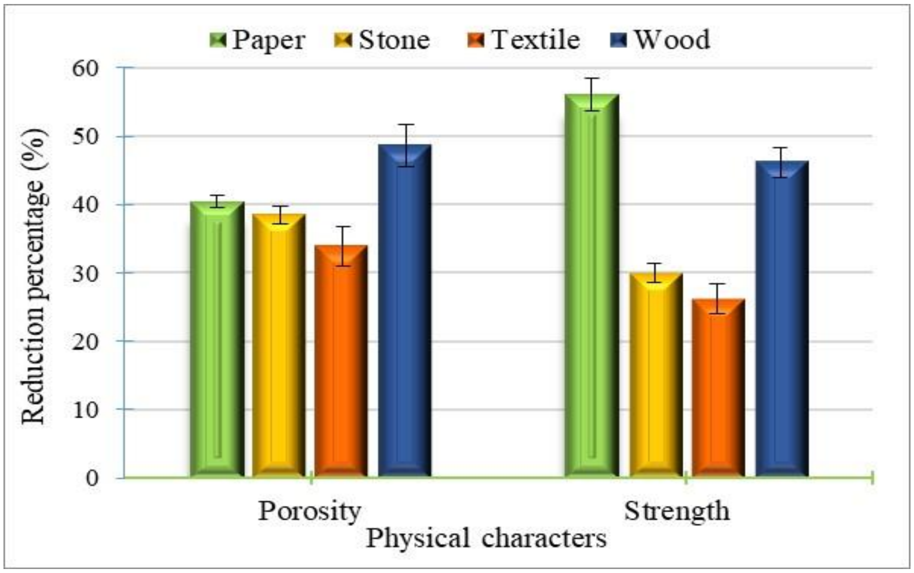

Fig. S4. Reduction percentages in the physical characteristics of paper, stone, textile and wood caused by *A. fumigatus* NMEC-PSTW.1 growth. The bars represent the standard error (n = 3).

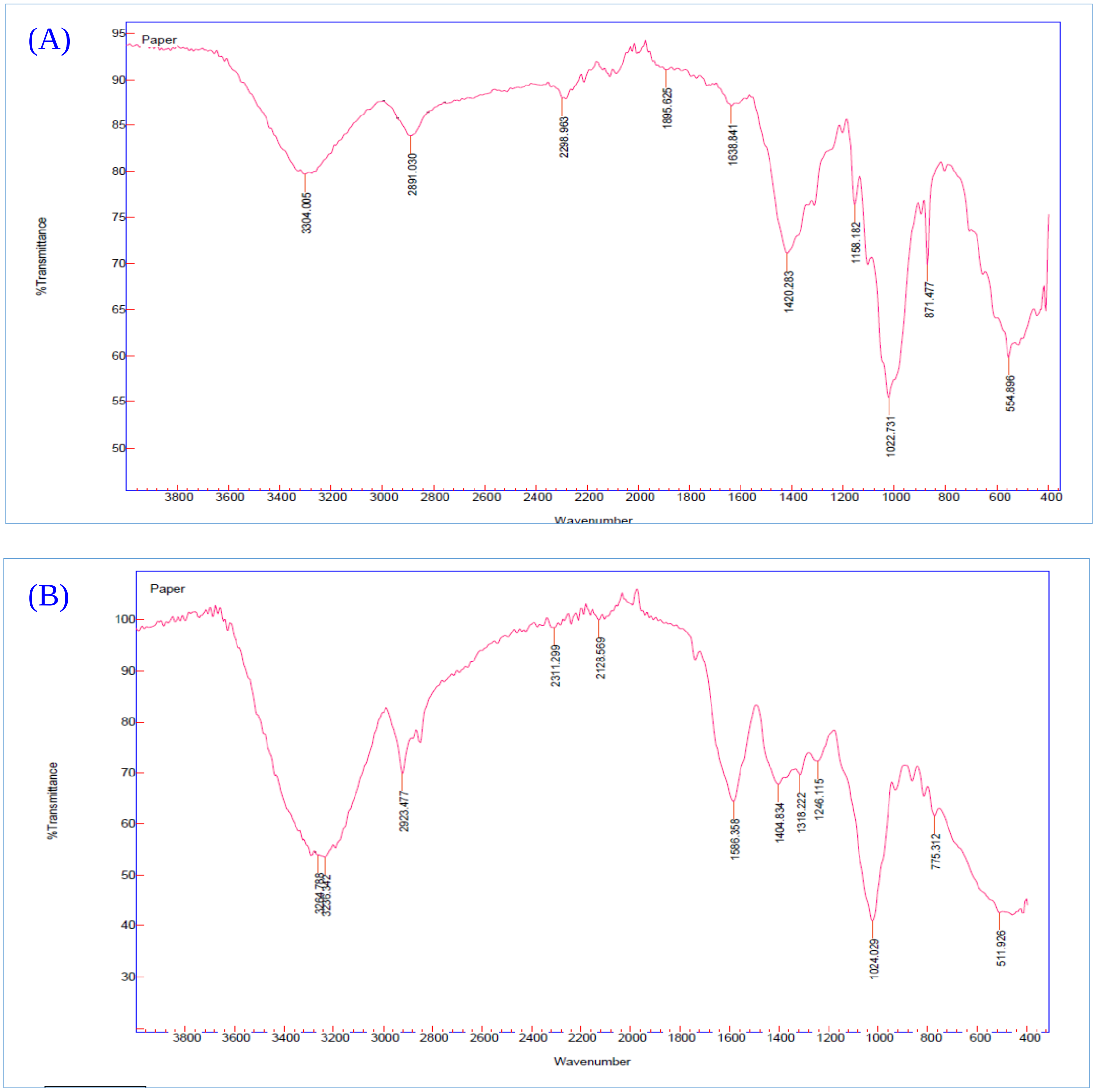

Fig. S5A. FTIR analysis of paper cubes. (A) noncolonized cube (control) and (B) cubes inoculated with *A. fumigatus* NMEC-PSTW.1.

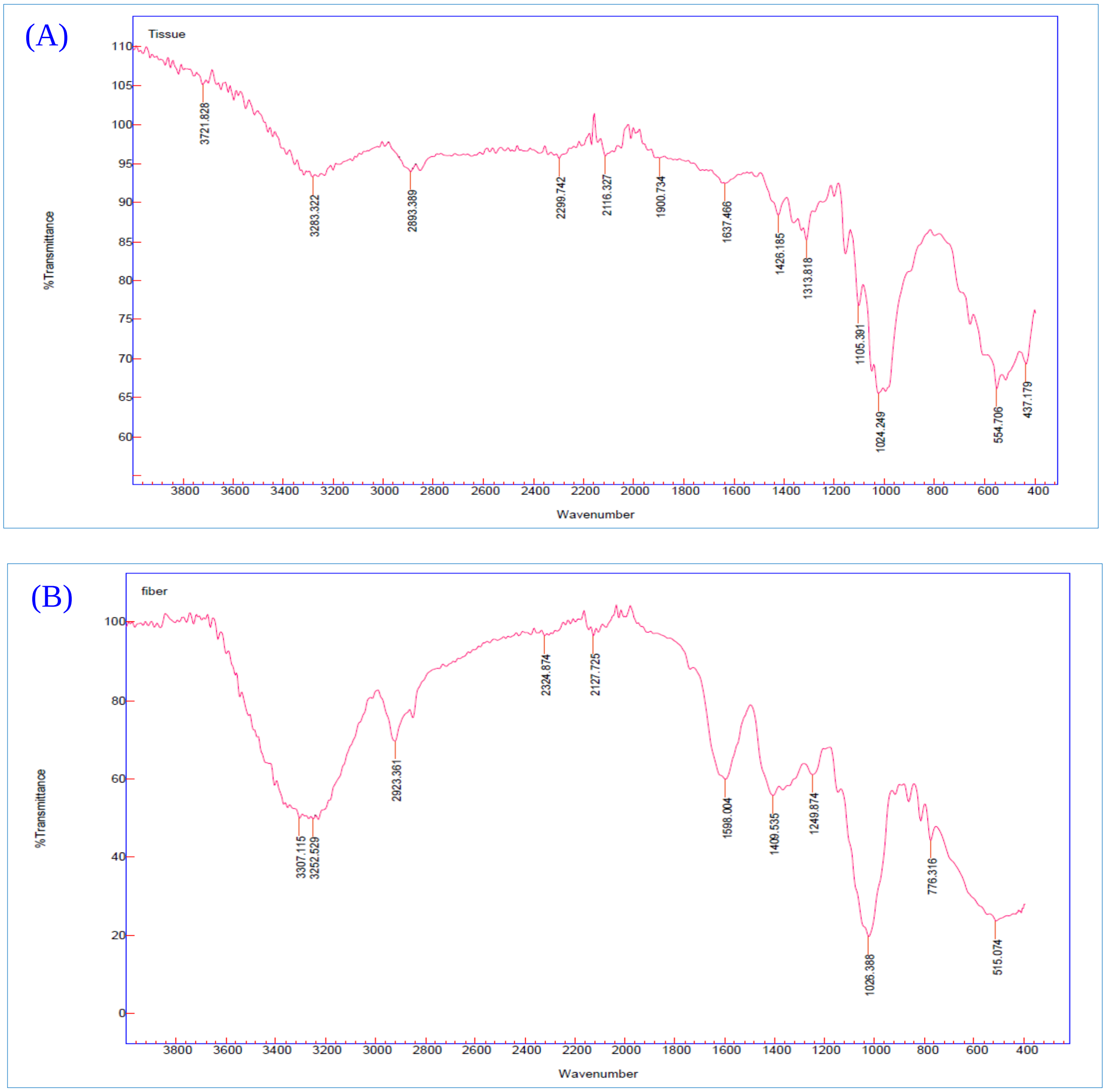

Fig. S5B. FTIR analysis of textile cubes. (A) noncolonized cube (control) and (B) cubes inoculated with *A. fumigatus* NMEC-PSTW.1.

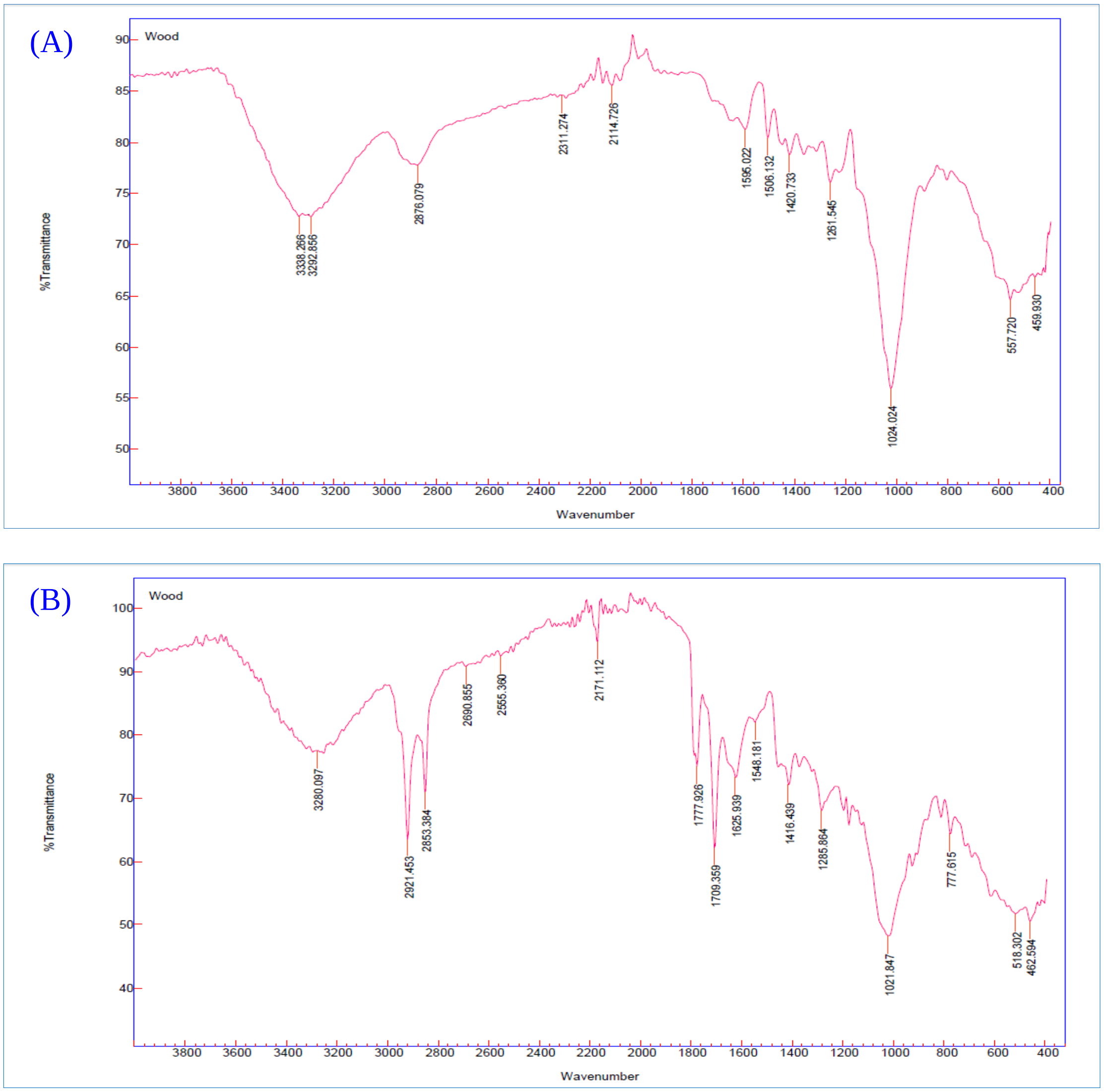

Fig. S5C. FTIR analysis of wood cubes. (A) noncolonized cube (control) and (B) cubes inoculated with *A. fumigatus* NMEC-PSTW.1.
